# Relative Performance of Volume of Distribution Prediction Methods for Lipophilic Drugs with Uncertainty in LogP Value

**DOI:** 10.1007/s11095-024-03703-4

**Published:** 2024-05-08

**Authors:** Ana L. Coutinho, Rodrigo Cristofoletti, Fang Wu, Abdullah Al Shoyaib, Jennifer Dressman, James E. Polli

**Affiliations:** 1 Department of Pharmaceutical Sciences, University of Maryland School of Pharmacy, 20 Penn Street, Room 623, HSF2 Building, Baltimore, MD 21201 USA; 2 Department of Pharmaceutics, Center for Pharmacometrics and Systems Pharmacology, College of Pharmacy, University of Florida, Orlando, FL USA; 3 Office of Generic Drugs, Food and Drug Administration, White Oak, MD USA; 4 Fraunhofer Institute of Translational Medicine and Pharmacology, Theodor-Stern-Kai 7, 60596 Frankfurt am Main, Germany

**Keywords:** lipophilicity, logP, volume of distribution

## Abstract

**Purpose:**

The goal was to assess, for lipophilic drugs, the impact of logP on human volume of distribution at steady-state (VD_ss_) predictions, including intermediate fut and Kp values, from six methods: Oie-Tozer, Rodgers-Rowland (tissue-specific Kp and only muscle Kp), GastroPlus, Korzekwa-Nagar, and TCM-New.

**Method:**

A sensitivity analysis with focus on logP was conducted by keeping pKa and fup constant for each of four drugs, while varying logP. VD_ss_ was also calculated for the specific literature logP values. Error prediction analysis was conducted by analyzing prediction errors by source of logP values, drug, and overall values.

**Results:**

The Rodgers-Rowland methods were highly sensitive to logP values, followed by GastroPlus and Korzekwa-Nagar. The Oie-Tozer and TCM-New methods were only modestly sensitive to logP. Hence, the relative performance of these methods depended upon the source of logP value. As logP values increased, TCM-New and Oie-Tozer were the most accurate methods. TCM-New was the only method that was accurate regardless of logP value source. Oie-Tozer provided accurate predictions for griseofulvin, posaconazole, and isavuconazole; GastroPlus for itraconazole and isavuconazole; Korzekwa-Nagar for posaconazole; and TCM-New for griseofulvin, posaconazole, and isavuconazole. Both Rodgers-Rowland methods provided inaccurate predictions due to the overprediction of VD_ss_.

**Conclusions:**

TCM-New was the most accurate prediction of human VD_ss_ across four drugs and three logP sources, followed by Oie-Tozer. TCM-New showed to be the best method for VD_ss_ prediction of highly lipophilic drugs, suggesting BPR as a favorable surrogate for drug partitioning in the tissues, and which avoids the use of fup.

**Supplementary Information:**

The online version contains supplementary material available at 10.1007/s11095-024-03703-4.

## Introduction

Volume of distribution at steady-state (VD_ss_) is a key pharmacokinetic parameter that, in conjunction with clearance, impacts half-life and dosing regimen. VD_ss_ prediction is important in drug discovery and development, such as designing first-in-human studies, pediatric dose prediction or dose extrapolation, and therapeutic drug monitoring for narrow therapeutic index drugs. Drug distribution is modulated by the ratio of drug present in tissues, blood, and plasma. The Oie-Tozer, Rodgers-Rowland, GastroPlus, Korzekwa-Nagar, and TCM-New models aim to predict VDss by, in part, considering the mechanisms behind drug partition.

Lipophilicity and ionization are important drug physicochemical properties that affect VD_ss_. They impact drug permeability and binding to cell membranes, intracellular and extracellular protein binding, and affinity for enzymes and cell transporters [1–4]. A prior global sensitivity analysis showed that, among parameters such as log of the drug partition coefficient (logP), pKa, fraction of drug unbound in plasma (fup), and drug blood-to-plasma ratio (BPR), logP was the most influential parameter in determining drug tissue-to-plasma partition coefficient (Kp) for neutral and weakly basic drugs [5]. Among physicochemical parameters such as pKa, molecular weight (MW), logP, intrinsic solubility, number of hydrogen donors, number of hydrogen acceptors, and polar surface area, logP was strongly correlated with an increase *in vivo* rat Kp for neutral and basic drugs [6]. LogP has an even larger determination on *in vivo* rat adipose tissue Kp for neutral and basic drugs. In addition to affecting Kp, logP is also essential to calculate fraction of drug unbound in plasma (fut) predictions in the Oie-Tozer method and fraction unbound in microsomes (f_um_) in the Korzekwa-Nagar method [7, 8].

Human adipose tissue Kp has been observed to plateau for highly lipophilic drugs [9]. Interestingly, Rodgers and Rowland acknowledge that their method for Kp and VD_ss_ predictions may overestimate these parameters for compounds with high logP (e.g., logP > 3) [10]. This potential bias appears to be recognized but underappreciated. Underpinning this underappreciation is, we believe, the lack of reliable drug logP values, as logP is required for the Oie-Tozer, Rodgers-Rowland, GastroPlus, Korzekwa-Nagar and TCM-New methods as well.

Berry *et al*. (2011) reported that predicted VD_ss_ using Rodgers-Rowland resulted in more than fourfold overprediction for compounds with logP greater than 3.5, with some compounds showing overprediction of about 100-fold [11]. Chan *et al*. (2018) found that Rodgers-Rowland also overpredicted VD_ss_ for lipophilic neutral and basic compounds. This method became less reliable when logP was high, particularly when logP > 4, even when experimental logP was obtained from literature resources [12]. Previous work has shown that Rodgers-Rowland overpredicts Kp when fup is not adjusted. Highly lipophilic drugs also suffer from challenges in accurate fup measurements which may substantially affect VDss predictions [10, 13].

Partitioning into octanol, vegetable oil, or other organic phases may not adequately represent the drug partition into the classes of neutral lipids present in plasma and tissues (e.g., triglycerides, diglycerides, monoglycerides, cholesterol) [11, 14]. Since triglycerides are the most predominant lipid in the adipose tissue, the use of vegetable oil:water partition in place of octanol:water partition has been considered as vegetable oil is more closely related structurally to triglycerides than octanol. However, using vegetable oil may pose other issues since there are variability in oil composition that is not seen with octanol [14]. Among six methods examined here, the TCM-New is the only method that includes vegetable oil:water partition in VD_ss_ predictions, incidentally, in addition to octanol:water logP [13].

The accuracy and even availability of logP values is an issue, as there is a lack of experimentally determined logP values in the literature [15]. We have also questioned the accuracy of reported logP value for high logP compounds, particularly computationally estimated logP values [15]. Others have indicated that highly lipophilic drugs often do not have experimentally measured logP values [16].

The overall goal of this study was to assess the impact of drug logP on VD_ss_ predictions, including intermediate fut and Kp values, from six methods: Oie-Tozer, Rodgers-Rowland (both tissue-specific Kp, and muscle Kp), GastroPlus, Korzekwa-Nagar, and TCM-New. We aimed to determine how variation in logP can impact variation in VD_ss_ predictions, as well as which methods are most sensitive to changes in the source of logP values. In particular, one objective was to compare the sensitivity of these six methods over a range of logP values when logP values are high. Another objective was to compare VD_ss_ prediction errors for specific drug logP values. The four drugs in this study were chosen since they are lipophilic, and each has a wide range of experimental and in silico logP values reported in the literature.

## Material and Methods

### Overall Study Approach

**Fig. 1:**
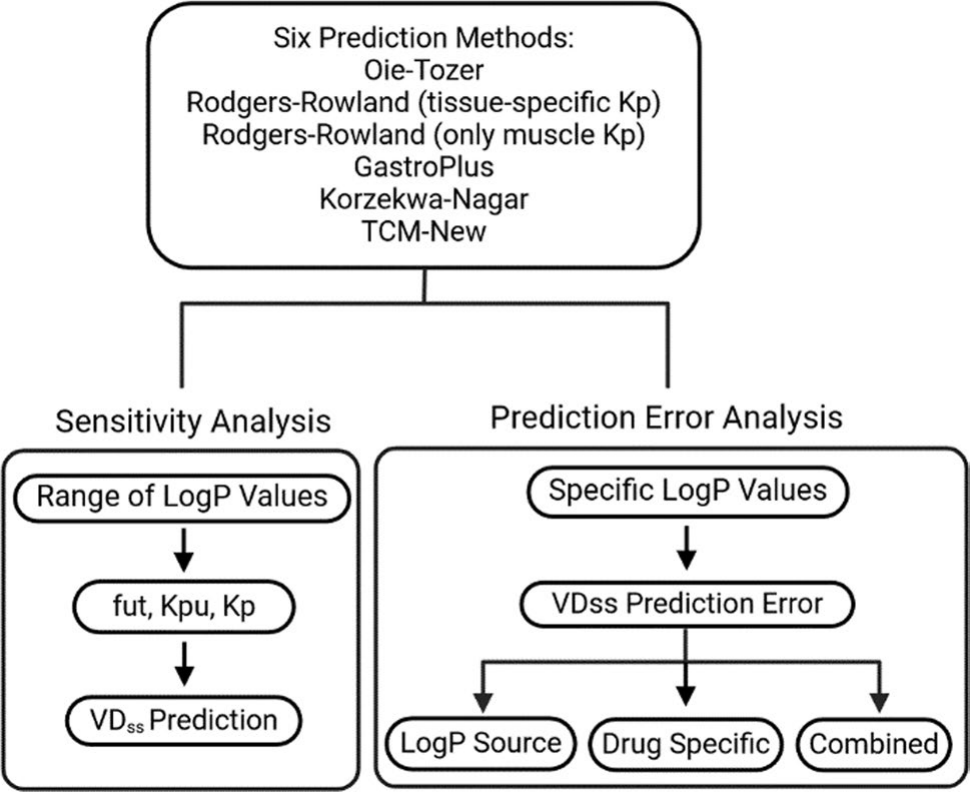
Workflow here in performing each sensitivity analysis, as well as prediction error analysis. Objective one concerned sensitivity analysis over a range of logP values. Objective two concerned prediction error analysis using specific logP values. Physicochemical properties for each method are listed in Table II, including logP. VD_ss_ is estimated here via Oie-Tozer, Rodgers-Roland, GastroPlus, Korzekwa-Nagar, and TCM-New methods.

A workflow of our analysis is shown in Fig. 1. This study compared six prediction methods. The assumptions of each method and their intermediate parameters for VD_ss_ prediction are shown in Table I.

**Table I:** Model Assumptions and Required Drug Specific Data for Oie-Tozer, Rodgers-Rowland, GastroPlus, Korzekwa-Nagar, and TCM-New Methods

Volume of distribution method	Intermediate parameter	Information for intermediate parameter estimation	Model assumptions
Oie-Tozer [6, 15–17]	fut (i.e., Eq. 3)	pKa, logP, fup	fut is the same in all tissues. Human fut is the average animal fut across species. R_e/i_ is the same for all binding proteins in the extracellular fluid and plasma in both animals and humans
Rodgers-Rowland [8, 18, 19]	Kpu (i.e., Eq. 4)	fup, pKa, logP (or P)	Note: $Kpu=Kp\bullet fup$ Drugs dissolved in intra- and extra-cellular tissue water and unbound unionized drug will partition into lipids (i.e., neutral lipids and phospholipids) within tissue cells. In the extracellular space, neutral and weakly acidic drugs will bind only to albumin. Weakly acidic drugs bind preferentially to albumin, and neutral drugs will bind to lipoprotein. Both albumin and lipoprotein are abundant in the extracellular space, so they will have a great effect on tissue distribution of weakly basic and neutral drugs. Calculated rat Kpu is assumed to be the same as human Kpu
GastroPlus (perfusion limited model)	Kp (i.e., Eq. 4)	fup, pKa, logP Neutral and weakly basic drugs follow the same Rodgers-Rowland equation for Kp	Note: $Kpu=Kp\bullet fup$ Same model assumptions as the Rodgers-Rowland method. Additionally, in light of Perfusion Limited Model, the concentration of unbound unionized drug is the same in the extracellular, intracellular, and plasma spaces. Instant drug equilibrium is reached in the intracellular and extracellular spaces
Korzekwa-Nagar [8, 20]	L*K_L_ (i.e. Equation 12)	# of H-bond donors and acceptors, number of SO and NO_2_ groups, logp, pK_a,a_, pK_a,b_	Tissue-lipid partitioning is represented by f_um_. Assumes two compartments: tissue and plasma. Plasma proteins exist in plasma and tissue spaces. Unbound drug in plasma is at equilibrium with drug bound to plasma proteins and free drug in the tissue. Free drug in tissue can bind to proteins and lipids. Only unionized drugs bind to neutral lipids
TCM-New [13]	None	NA	BPR is a representative of drug partitioning into tissues and plasma. Blood contains cells and plasma (extracellular fluid) that can be comparable to tissues. The membrane of red blood cells regulates drug distribution similarly to tissue cell membrane

Griseofulvin, itraconazole, posaconazole, and isavuconazole were chosen because they are lipophilic (experimental logP > 3) and have a wide range of reported logP values in the literature (i.e., a range of at least one logP unit). In addition, these drugs have literature human VD_ss_ from clinical studies using intravenous (IV) administration. Table II provides the logP data based on ADMET Predictor (Simulations Plus, version 10.4), literature logP, and HPLC-based logP. The logP values for griseofulvin span 2.411 to 3.53, itraconazole span 4.893 to 6.888, posaconazole span 4.405 to 6.716, and isavuconazole span 3.56 to 4.934. ADMET Predictor was used to calculate drug pKa and BPR.

**Table II:** Drug Physicochemical Properties in the Literature and Predicted by ADMET Predictor

	ADMET Predictor			Reported		
Drugs	LogP	pKa	BPR	fup	Literature logP	HPLC-based logP [15]
Griseofulvin	2.511	N/A	0.776	0.16^a^ [21]	3.53^f^ [22]	3.566
Itraconazole	4.893	4.57 (base)	0.665	0.002^b−d^ [23–25]	5.66^ g^ [23]	6.888
Posaconazole	4.405	4.67 (base)	0.668	0.02^b,e^ [25–27]	5.36^f^ [27]	6.716
Isavuconazole	3.619	3.28 (base)	0.737	0.01^b,c^ [25, 28]	3.56^ g^ [29]	4.934

Method of experimental fup and logP: ^a^ fup equilibrium dialysis; ^b^ fup method unknown; ^c^ FDA drug package insert (method unknown); ^d^ fup dilution; ^e^ ultrafiltration; ^f^ logP in silico; ^g^ logP shake-flask or slow-stirring method

All methods used to predict VD_ss_ focus on drug partitioning in plasma, extracellular fluid, and/or body tissues; none consider active drug transport. Oie-Tozer, Rowland-Rodgers, GastroPlus, and TCM-New methods do not take into consideration lysosomal trapping, microsomal partitioning, or active transport [5, 10, 13, 18, 30]. Korzekwa-Nagar uses microsomal partitioning as a surrogate for general cell membrane partitioning [8].

Objective one involved sensitivity analysis of fut, Kp, and VD_ss_ for a range of logP values. A sensitivity analysis with focus on logP was conducted with pKa and fup kept constant for each drug, while logP was varied. An analysis of how fut, Kp, and VD_ss_ changed based on logP was conducted at every 0.5 logP units within the range of reported logP values (e.g., logP values 2, 2.5, 3, 3.5, 4, and 4.5 for griseofulvin).

Objective two involved VD_ss_ prediction errors for specific logP values. VD_ss_ was calculated from reported logP values to assess VD_ss_ prediction performance. Reported logP values were sourced from ADMET Predictor, literature, and HPLC-based literature. In general, for each drug, logP value tended to rank-order as follows: ADMET Predictor < literature < HPLC-based literature. The assessment of the VD_ss_ prediction errors was conducted three ways: first, by grouping VD_ss_ predictions by logP source; second, by grouping VD_ss_ predictions by drug; and third, combining all VD_ss_ prediction across all drugs and logP sources.

All VD_ss_ predictions were then compared to VD_ss_ reported from clinical studies from IV administration, as well as to the calculated VD_ss_ from non-compartmental (NCA) and compartmental analysis.

Intermediate and VD_ss_ predictions using Oie-Tozer, both Rodgers-Rowland, Korzekwa-Nagar, and TCM-New methods were calculated using Excel (Microsoft, Version 2308). Kp and VD_ss_ using the GastroPlus method was calculated using the software GastroPlus (Simulations Plus, Version 10.4). NCA and compartmental analysis were performed using PKPlus software (Simulations Plus, Version 10.4).

As noted above, the four drugs were chosen since they are lipophilic, and each has a wide range of experimental and in silico logP values reported in the literature. Of note, itraconazole, isavuconazole, and posaconazole are azoles, but the azole fragment is a small component to each drug’s structure. Isavuconazole (MW = 437.47 g/mol and logP = 4.934) substantially differs structurally from posaconazole (MW = 700.5 g/mol and logP = 6.716) and itraconazole (MW = 705.6 g/moland logP = 6.814). As methods and results show, findings about itraconazole and posaconazole, including the relative predictive performance of the six methods for these two drugs, are different, in part since itraconazole and posaconazole have a tenfold difference in fup and over threefold difference in VD_ss_.

### Estimation of VD_ss_ Using the Oie-Tozer Equation

In Oie-Tozer, a drug can be in six different forms/spaces in the body: bound and unbound to proteins in plasma; bound and unbound to proteins in the extracellular fluid; and bound and unbound to tissue intracellularly [30, 31]. In the Oie-Tozer equation (Eq. 1),

1 $$VDss=V_p+fup\cdot V_e+R_{e/i}\cdot V_p\left(1-fup\right)+V_r\cdot\frac{fup}{fut}$$

where V_p_ is the plasma volume (0.0436 L/kg), fup is the fraction of drug unbound in plasma, V_e_ is the volume of extracellular fluid not considering the plasma volume (0.151 L/kg), R_e/i_ is the ratio of binding proteins in the extracellular fluid to the binding proteins in plasma (value 1.4), V_r_ is the volume that drug distributes to minus the extracellular fluid not considering the plasma volume (0.38 L/kg), and fut is the fraction of drug unbound in the tissues [19, 30]. Oie-Tozer assumptions are that drugs diffuse freely between plasma, extracellular fluid, and tissues; fut is the same across all tissues in the body; and R_e/i_ is the same for all binding proteins in the extracellular space and plasma (Table II) [30, 31].

Drug fut in humans cannot readily be measured experimentally. Obtaining necessary human tissue and determining drug unbound is not practically feasible. Hence, animal fut is sometimes determined, although it has economic and ethical limitations. Alternatively, a non-invasive approach to estimate human fut based on the model developed by Lombardo *et al*. 2004 [7] was used:

2 $${\text{log}}\left(fut\right)=0.0080-0.2294logP-0.9311{f}_{i\left(7.4\right)}+ 0.8885log{fu}_{p}$$

where f_i(7.4)_ is drug fraction ionized at pH 7.4. f_i(7.4)_ was calculated from drug pKa and the Henderson-Hasselbalch equation. It is noted that although Lombardo *et al*. used logD, Eq. 2 employs logP since all drugs investigated are neutral at pH 7.4. Lombardo *et al*. developed their model using 120 drugs in a training set. In this work, fut was calculated for itraconazole, griseofulvin, posaconazole and isavuconazole fut across the respective range of logP. Values for fup were held constant and these are listed in Table II. fup values from the literature were examined to rule out suspicious values [13]. Then, each fut was used to estimate VD_ss_ using Eq. 1.

### Estimation of Volume of Distribution Using Rodgers-Rowland Equation and Tissue-Specific Kp

Unbound drug tissue-to-plasma water partition coefficient (Kpu) was used, as an intermediate parameter, to estimate the volume of distribution at steady-state of unbound drug (Vu_ss_) using the Rodgers-Rowland method [10, 32, 33]:

3 $$\begin{array}{c}Kpu=\frac{X\cdot f_{IW}}Y+f_{EW}+\left[\frac{P\cdot f_{NL}+\left(0.3P+0.7\right){\cdot f}_{NP}}Y\right]+\\\left[\left(\frac1{fup}-1-\left(P\cdot f_{NL,P}+\left(0.3P+0.7\right){\cdot f}_{NP,P}\right)\right)\frac{{\left[PR\right]}_T}{{\left[PR\right]}_P}\right]\end{array}$$

where *f* is the fractional tissue volume; P is the n-octanol:water partition coefficient [vegetable oil:water partition coefficient can be used for the adipose tissue]; and PR is the concentration of acidic phospholipids and extracellular albumin (for weakly basic drugs) or lipoprotein (for neutral drugs). Subscripts are IW for intracellular water, EW for extracellular water, NL for neutral lipids, NP for neutral phospholipids, T for tissue, and P for plasma [32]. For neutral drugs like griseofulvin, X = Y = 1; for weakly basic drugs like itraconazole, isavuconazole, and posaconazole, $X=1+{10}^{pKa-{pH}_{IW}}$ and $Y=1+{10}^{pKa-{pH}_{P}}$. Values for pH_IW_, pH_p_, f_NL,P_, f_NP,P_ were 7.22, 7.4, 0.0023, and 0.0013, respectively [18]. These tissue specific parameter values are listed in Table III.

**Table III:** Input Parameters for Kpu Predictions in Rat to be Used for Rodgers-Rowland Vu_ss_ Calculations. Tissue Volumes are for a 70 kg Human [10, 19, 32–35]

		Residual blood adjusted fractional tissue volumes				Tissue-to-plasma [PR]_T_/[PR]_P_	
Tissue	Volume (L)	Neutral lipid (f_NL_)	Neutral phospholipid (f_NL_)	Extracellular water (f_NL_)	Intracellular water (f_NL_)	Albumin ratio	Lipoprotein ratio
Adipose	10.43	0.0016	0.853	0.135	0.017	0.049	0.068
Bone	9.07	0.0174	0.0016	0.1	0.346	0.1	0.05
Brain	1.49	0.0391	0.0015	0.162	0.62	0.048	0.041
Gut	1.85	0.0375	0.0124	0.282	0.475	0.158	0.141
Heart	0.31	0.0135	0.0106	0.32	0.456	0.157	0.16
Kidney	0.31	0.0121	0.024	0.273	0.483	0.13	0.137
Liver	2.52	0.0135	0.0238	0.161	0.573	0.086	0.161
Lung	0.92	0.0215	0.0123	0.336	0.446	0.212	0.168
Muscle	33.89	0.01	0.0072	0.118	0.63	0.064	0.059
Pancreas	0.21	0.0403	0.009	0.12	0.664	0.06	0.06
Skin	5.63	0.0603	0.0044	0.382	0.291	0.277	0.096
Spleen	0.2	0.0071	0.0107	0.207	0.579	0.097	0.207
Thymus	0.039	0.0168	0.0092	0.15	0.626	0.075	0.075

Drug specific parameters such as logP, pKa, BPR, and fup are shown in Table II. For each drug, Kpu was calculated for each of the 13 tissues over a range of logP. Equation 3 was used to calculate Kpu for neutral (griseofulvin) and weakly basic drugs (itraconazole, posaconazole, and isavuconazole). The only input difference is that for neutral drugs, the drug tissue-to-plasma input ([PR]_T_/[PR]_P_) was the lipoprotein ratio; for weakly basic drugs, it was the albumin ratio.

Both *in vivo* human and animal Kpu values are very limited, in part due to ethical considerations in generating human specific tissue composition data. Here, rat tissue physiological composition was applied to calculate human Kpu. The accuracy of rat-based estimates is uncertain (e.g., determined at steady-state in rats and for example do not account for tissue metabolism) [10, 36]. However, *in vivo* and calculated rat tissue Kpu have been shown to agree with human Vu_ss_ (or VD_ss_) [6, 10, 32, 33].

Then, human Vu_ss_ was calculated using the Rodgers and Rowland equation (Eq. 4) for each drug, over a range of logP values:

4 $${Vu}_{ss}=\frac{V_p}{fup}+\sum V_{T,i}\cdot{Kpu}_i$$

where V_p_ is the plasma volume, V_T,i_ is the volume of the *i*th tissue, and the Kpu_,i_ is the Kpu of the unbound drug in the *i*th tissue. The tissue volumes normalized by body weight for a 70 kg human, with the plasma volume set to 3.5 L, are presented in Table III [10, 19, 33, 34].

Although calculated in this work, Vu_ss_ is not commonly discussed in the literature, because the most commonly reported form of volume of distribution is a combination of bound and unbound drug (VD_ss_). Therefore, for the purpose of comparing VD_ss_ prediction methods, Vu_ss_ from Eq. 4 was converted to VD_ss_ using Eq. 5:

5 $${VD}_{ss}={Vu}_{ss}\cdot fup$$

### Estimation of Volume of Distribution Using Rodgers-Rowland Equation and Muscle Kp for All Tissues

The Rodgers-Rowland analysis described above employed 13 unique tissues. A second Rodgers-Rowland approach, using only muscle Kpu for all 13 tissues, simplifies the calculation of human Vu_ss_ across a range of logP values. Predicted muscle Kpu was calculated using Eq. 3 using the parameters listed in Tables I and III. Predicted human Vu_ss_ based only on muscle Kpu used Eq. 6, whereby muscle Kpu was multiplied by the sum the volume of the 13 tissues listed in Table III:

6 $${Vu}_{ss}=\frac{V_p}{f_{up}}+\sum\nolimits_1^{13}V_i\cdot{Kpu}_{muscle}$$

Vu_ss_ was converted to VD_ss_ using Eq. 5 for the reasons described above. *In vivo* rat muscle Kp has been denoted as a good predictor for Kp in other tissues; on the other hand, rat adipose tissue Kp was found to be the worst predictor of other tissue’s Kp (except stomach and pancreas) and human VD_ss_ [6, 9, 10].

### Estimation of Volume of Distribution Using Perfusion Limited Model in GastroPlus

Overall, the GastroPlus-based approach involved using the adjusted fup to calculate Kp. Kp was calculated using the original Rodgers-Rowland equation (Eq. 3). GastroPlus calculates VD_ss_ using Eq. 7,

7 $$V_{ss}=V_p+V_e\cdot E:P+\sum V_t\cdot{Kp}_t\left(1-{ER}_t\right)$$

where V_p_ is plasma volume, V_e_ is erythrocyte volume, E:P is drug erythrocyte to plasma concentration ratio (calculated from drug blood/plasma concentration ratio and hematocrit), V_t_ is specific tissue volume, Kp_t_ is the tissue-specific Kp, and ER_t_ is the extraction ratio for a given tissue [17]. Tissue volumes were set from the population estimates for age-related physiology (PEAR Physiology) in the GastroPlus PBPK module. The population was set to a 70-kg 30-year-old American male.

A parameter in Eq. 7 is Kp, which is generated by calculating Kpu using Eq. 3, and then converting it to Kp using Eq. 8.

8 $$Kp={Kp}_u\cdot{fu}_{p(adj)}$$

where fup_(adj)_ is the adjusted fup which is calculated according to Eq. 9:

9 $${fup}_{(adj)}= \frac{1}{{10}^{{logD}_{(7.4)}}\cdot\left(\frac{{V}_{lipid}}{{V}_{water}}\right)+1+\frac{1-fup}{fup}}$$

where V_lipid_ is the volume fraction of total neutral lipid and phospholipid in plasma, and V_water_ is the volume fraction of water in plasma.

GastroPlus calculates VD_ss_ using a modified approach of the original Rodgers-Rowland method. The original Rodgers-Rowland method involves two Kpu equations, one to calculate Kpu for neutral, acid, or weak bases, and another to calculate Kpu for moderate-to-strong bases. GastroPlus combines these two equations into a single equation to provide a continuous shift from albumin (neutral, acidic, and weakly basic drugs) to acidic phospholipids binding (moderate-strong bases) [37]. The combined equation is immaterial here since only neutral and weakly basic drugs (pKa 2–5) were evaluated in this work. That is, for the drugs studied, the GastroPlus Kpu equation is Eq. 3 (i.e., the same for Rodgers-Rowland equation) since neutral and weakly basic drugs have no positive charge in plasma (pH 7.4). In GastroPlus software, the method is named Rodgers-Single. All Kpu calculations employed the Perfusion Limited Tissue Model since the drugs are lipophilic and mostly neutral at physiological pH. In this model, drug Kpu is assumed to be reached instantaneously. GastroPlus software aims to avoid overprediction of Kpu for highly lipophilic drugs by adjusting drug fup [38].

The adjusted fup is described by Eq. 9. Experimental fup values for the four model drugs are presented in Table II.

### Estimation of Volume of Distribution Using Korzekwa-Nagar

Korzekwa and Nagar developed a VD_ss_ prediction method that, like the Oie-Tozer method, involves drug distribution to the intracellular, extracellular, and plasma spaces [8, 20]. In this model, unbound drug partitioning to the microsome mimics drug partitioning to the cell membrane during drug distribution. The unbound drug fraction that binds to microsomal membrane (f_um_) represents the drug fraction that partition to the tissue lipids.

The Korzekwa-Nagar method predicts VD_ss_ by using immediate drug parameters fup and f_um_. VD_ss_ is calculated using Eq. 10 [20]:

10 $${VD}_{SS}=V_p+V_t\cdot f_{up}+V_t\cdot R_1\left(1-f_{up}\right)+f_{up}\left(a\left(\frac{1-f_{um}}{f_{um}}\right)+b\right)$$

where V_p_ is plasma volume (0.043 L/kg), V_t_ is tissue volume (0.557 L/kg), and R_1_ is the ratio of plasma protein concentration in tissue and plasma (0.116 for neutral drugs). Constants a and b were 20 and 0.76, respectively. In the model development, drugs with fup < 0.005 were excluded [20]. f_um_ here was predicted using the approach developed by Korzekwa and Nagar (Eqs. 11 and 12) [8, 20].

11 $$\frac{1-f_{um}}{f_{um}}=L\cdot K_L$$

12 $$\begin{array}{c}Log\left(L\cdot K_L\right)=Log\\\left(10^{const1+a1\bullet logP+d1\bullet dipole+e\bullet SO+f\cdot{NO}_2}+10^{const2+a2\cdot logP+b2\cdot acc+c2\cdot don+e\cdot SO+f{\cdot NO}_2+{pK}_{a,b}-7.4}+10^{const3+b3\cdot acc+e\cdot SO+f{\cdot NO}_2+{pK}_{a,a}-7.4}\right)\\-logP\left(1+10^{{pK}_{a,b}-7.4}\right)-\text{log}\left(1+10^{7.4-{pK}_{a,a}}\right)\end{array}$$

where K_L_ is the lipid binding constant, L is the lipid concentration, acc is the number of hydrogen bond acceptors, don is the number of hydrogen bond donors, SO is number of SO groups, NO_2_ is the number of NO_2_ groups, pk_a,a_ is the acidic pKa, and pk_a,b_ is the basic pKa. Const, a, b, c, d, e, and f values are shown in Table S1 in Supplementary Information [8]. With the exception of logP and dipole moment, physicochemical parameters were predicted by ADMET Predictor and shown Table S2. Dipole moment was calculated using Molecular Operating Environment (MOE v2018.0101) software (Chemical Computing Group; Montreal, Canada). Final dipole moments were obtained from the “dipole” in property calculated though the MOE database properties capabilities.

### Estimation of Volume of Distribution Using New Tissue Composition-Based Model (TCM-New)

A new tissue composition model (TCM-New) for VD_ss_ prediction was recently developed to improve on the Rodgers-Rowland and GastroPlus methodologies, especially for neutral drugs [13]. Rodgers-Rowland and Poulin-Theil methods have shown the importance of BPR in predicting VD_ss_ of ionized basic drugs [5, 6, 13, 18, 30]. TCM-New expands the use of BPR for the VD_ss_ prediction of neutral drugs. This method focuses on membrane permeation as a key element in drug distribution to the intracellular space. The red blood cell membrane is thought to provide a similar environment that regulates drug access to the intracellular space. In addition, blood contains elements such as cells and plasma which correlates to cells and interstitial fluid in tissues. Therefore, BPR may predict drug partitioning into tissues as BRP is calculated as the ratio of drug concentration in blood over drug concentration in plasma.

Human VD_ss_ using the TCM-New method was calculated using Eq. 13 [13],

13 $${VD}_{ss}=BPR\times \frac{\left({IW}_{WB}\right)+\left({EW}_{WB}\right)+[{10}^{{logP}_{oil:buffer}}\times \left({NL}_{WB-all tissues}+\frac{{NLP}_{Blood}}{5}\right)]}{\left({IW}_{Blood}\right)+\left({EW}_{Blood}\right)+[{10}^{{logP}_{n-octanol:buffer}}\times \left({NL}_{Blood}+{NLP}_{Blood}\right)]}+{V}_{plasma}$$

where RBP is the blood-to-plasma ratio, IW_BW_ is the fractional content of intracellular water in the whole body (0.48 L/kg), EW_WB_ is the fractional content of extracellular water in the whole body (0.22 L/kg), NL_WB-all tissue_ is the fraction of neutral lipids equivalent for all tissues (sum of non-adipose and adipose tissues, 0.149 L/kg), NLP_Blood_ is the fraction of lipoproteins that are equivalent to neutral lipids in blood (0.00075 L/kg), IW_Blood_ is the intracellular water in the blood (0.29 L/kg), EW_Blood_ is the extracellular water in the blood (0.53 L/kg), NL_Blood_ is the sum of all neutral lipids equivalent in the blood (0.004 L/kg), NLP_Blood_ is the fraction of lipoproteins that are equivalent to neutral lipids in blood (0.00075 L/kg), and V_plasma_ is the plasma volume (0.04 L/kg). BPR values are shown in Table II. Calculations here assumed a 70 kg human. LogP_n-octanol:buffer_ refers to the drug partition between n-octanol and buffer and represents logP value regardless of method to generate the value. LogP_oil:buffer_ refers to the drug partition between vegetable oil and buffer. LogP_oil:buffer_ is not usually available in the literature but calculated from logP_n-octanol:buffer_ using Eq. 14.

14 $${LogP}_{oil:buffer}=1.099\times {logP}_{n-octanol:buffer}-1.31$$

### Calculation of Volume of Distribution using Non-Compartmental (NCA) and Compartmental Analysis

Human pharmacokinetic concentrations *versus* time profiles after IV administration from the literature were digitized using DigIt software (Simulations Plus, Version 10.4). NCA and compartmental analyses were performed using the PKPlus module within the GastroPlus software (Simulations Plus, Version 9.8.2). IV data references are listed in Table IV. One-, two-, and three-compartment models were fitted to the IV profile which was averaged across subjects.

**Table IV:** Volume of Distribution Reported from Human Studies (and CV%) and Calculated Here Using Non-compartmental (NCA) and Compartmental Analysis. Human Studies Employed IV Administration. VD_ss_ from Compartmental Analysis is the Sum of Central and Peripheral Compartments. Griseofulvin, Posaconazole, and Isavuconazole Followed a Two-Compartment Model, and Itraconazole Followed a Three-Compartment Model

	Literature		Computed in this study	
Drugs	Human VD_ss_ (L)	Human VD_ss_ reference	NCA VD_ss_ (L)	Compartmental analysis VD_ss_ (L)
Griseofulvin	107^a^ (6.7%)	[39]	99.9	112.1
Itraconazole	800^b^ (23.6%)	[40]	543.6	578.9
Posaconazole	294^c^ (39%)	[41]	240.1	250.4
Isavuconazole	304^d^ (28.5%)	[42]	272.1	295.2

Type of volume of distribution calculation: ^a^ VD_ss_ = sum of volume of distribution from all compartments; ^b^ Vd_area_ (volume of distribution in the elimination phase); ^c^ V_z_ (volume of distribution during the terminal phase); and ^d^ VD_ss_ = [(Dose * AUMC/AUC^2^) – (dose * T/2*AUC)]

VD_ss_ from NCA and compartmental analysis were compared to VD_ss_ from predictions using Oie-Tozer, Rodgers-Rowland (tissue-specific and only muscle Kp), Korzekwa-Nagar, and TCM-New, as well as to the reported volume of distribution from the literature IV data.. The sum of all volume of distribution from compartmental analysis (central and peripheral compartment) is denoted VD_ss_. NCA VD_ss_ was calculated as the product of mean residence time at infinity and clearance at steady-state.

In the compartmental analysis, the objective function weighting of 1/Yhat^2^ was applied and the best model was selected using Akaike Information Criterion. The two-compartment model best fit griseofulvin, posaconazole, and isavuconazole. The three-compartment model best fit itraconazole.. Except for itraconazole, all the other drugs had literature VD_ss_ very similar to the NCA and compartmental analysis results generated in this work. Itraconazole volume of distribution in the literature was reported as terminal phase volume of distribution which could explain the discrepancy with NCA and compartmental analysis results.

### Assessment of VD_ss_ Prediction Performance

VD_ss_ predictions were assessed by fold error, average fold error (AFE), and average absolute fold error (AAFE) compared to reported VD_ss_ from the literature.

15 $$average\;fold\;error= {10}^{\sum_{i=1}^{n}{\text{log}}\left(\frac{{VD}_{ss} predicted}{{VD}_{ss} observed}\right)/n}$$

16 $$absolute\;average\;fold\;error= {10}^{\sum_{i=1}^{n}abs({\text{log}}\left(\frac{{VD}_{ss} predicted}{{VD}_{ss} observed}\right))/n}$$

AFE indicates the bias of each prediction and AAFE indicates the precision (Eqs. 15 and 16). An AFE < 1 indicates an underprediction bias, and an AFE > 1 indicates overprediction bias. An AAFE of 1 indicates perfect prediction.

The assessment of the VD_ss_ predictions errors was conducted three ways: first, by grouping VD_ss_ predictions by logP source; second, by grouping VD_ss_ predictions by drug; and third, combining all VD_ss_ prediction across all drugs and logP sources. The motivation for applying these differing analyses is that the focus can sometimes be logP-source specific across multiple drugs (e.g., ADMET Predictor), can be drug-specific with multiple logP estimates (e.g., itraconazole), or be broadly applied across multiple drugs and multiple logP data sources.

## Results and Discussion

Results are presented firstly as sensitivity analysis, and secondly error prediction analysis, with an effort to assess the impact of high drug logP on VD_ss_ predictions, including intermediate fut and Kp values.

### Sensitivity Analysis: Impact of Drug logP on Fut and Kp Values

**Fig. 2:**
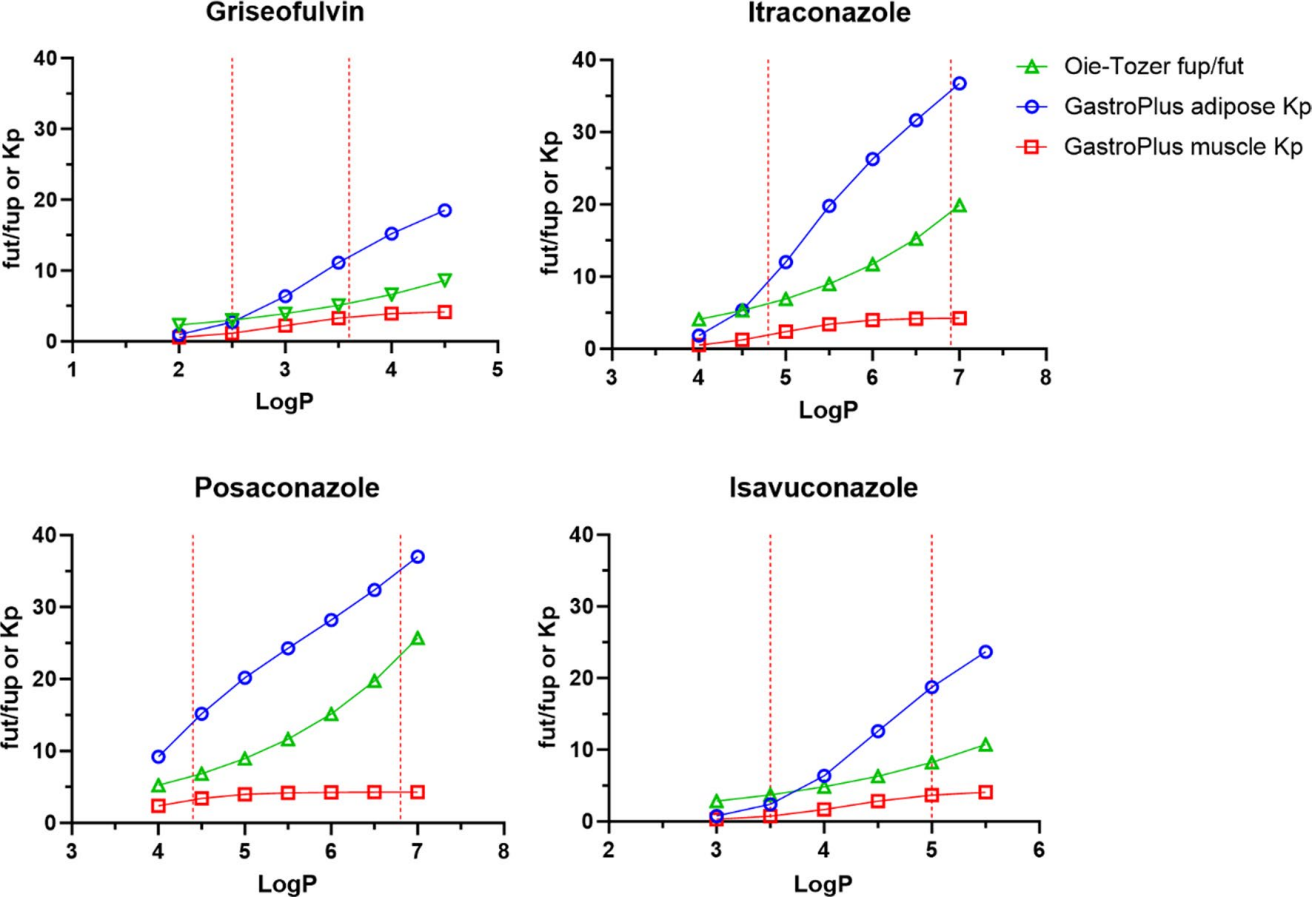
Griseofulvin, itraconazole, posaconazole, and isavuconazole fut/fup ratio (green triangle), adipose Kp (blue circle), and muscle Kp (red square) across a range of logP values. The ratio of fut/fup was calculated using fut calculated from Eq. 2 and fup from the literature. Adipose and muscle Kp were calculated using GastroPlus software. The lower and upper limits of reported and ADMET Predictor logP values are denoted as red dash lines in the X-axis (Table II).

**Fig. 3:**
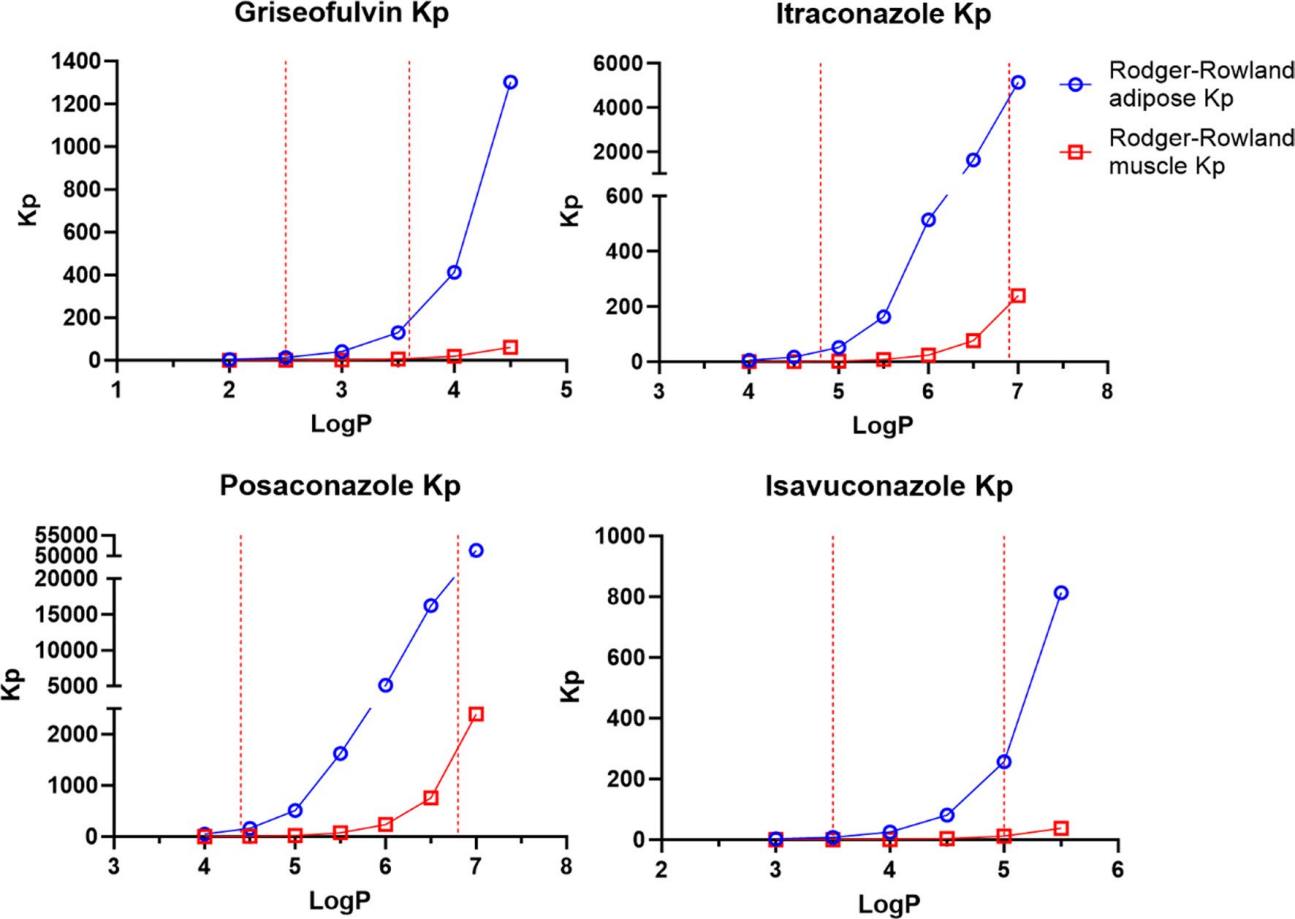
Griseofulvin, itraconazole, posaconazole, and isavuconazole adipose Kp (blue circle), and muscle Kp (red square) across a range of logP values. Kp was calculated by multiplying Kpu (Eq. 3) times fup. Kp results here in Fig. 4 are much larger than those in Fig. 3 (i.e., Rodgers-Rowland intermediates are much larger than Oie-Tozer and GastroPlus intermediates). The lower and upper limits of reported and ADMET Predictor logP values are denoted as red dash lines in the X-axis (Table II).

Griseofulvin, itraconazole, posaconazole, and isavuconazole fut and Kp were calculated for a range of logP values using the Oie-Tozer, Rodgers-Rowland (tissue-specific Kp and only muscle Kp), and the GastroPlus method. As expected, for each drug the Oie-Tozer fup/fut ratio and GastroPlus Kp increased as lipophilicity increased (Fig. 2). Among parameters impacted by logP, the three most sensitive parameters were adipose Kp > fup/fut ratio > muscle Kp. This finding was expected since drug partition to adipose tissue is highly mediated by logP, and muscle Kp has been shown to be less sensitive to logP [10]. In Eq. 2, fut is sensitive to logP, but fut represents a drug partition within different tissues in the body. Therefore, fut did not increase with logP to the same extent as adipose Kp. On the other hand, Rodgers-Rowland adipose Kp markedly grew with an increase in lipophilicity (Fig. 3), to an extent that appeared physiologically implausible. Rodgers-Rowland muscle Kp provided more plausible results for griseofulvin and isavuconazole. However, for the more lipophilic drugs itraconazole and posaconazole, the Rodgers-Rowland muscle Kp appeared excessively high. The values for Oie-Tozer fut, Rodgers-Rowland Kpu and Kp, and GastroPlus Kp predictions are shown in Tables S3-12.

### Sensitivity Analysis: Impact of Drug logP on VD_ss_ Predictions

The intermediate parameters fut and Kp were used to predict VD_ss_. Because of the uncertainty surrounding the logP estimates (Table II), VD_ss_ predictions were calculated for a range of logP values that encompassed the reported logP values. LogP values ranged from 2 to 4.5, 4 to 7, 4 to 7, and 3 to 5.5 for griseofulvin, itraconazole, posaconazole, and isavuconazole, respectively.

**Fig. 4:**
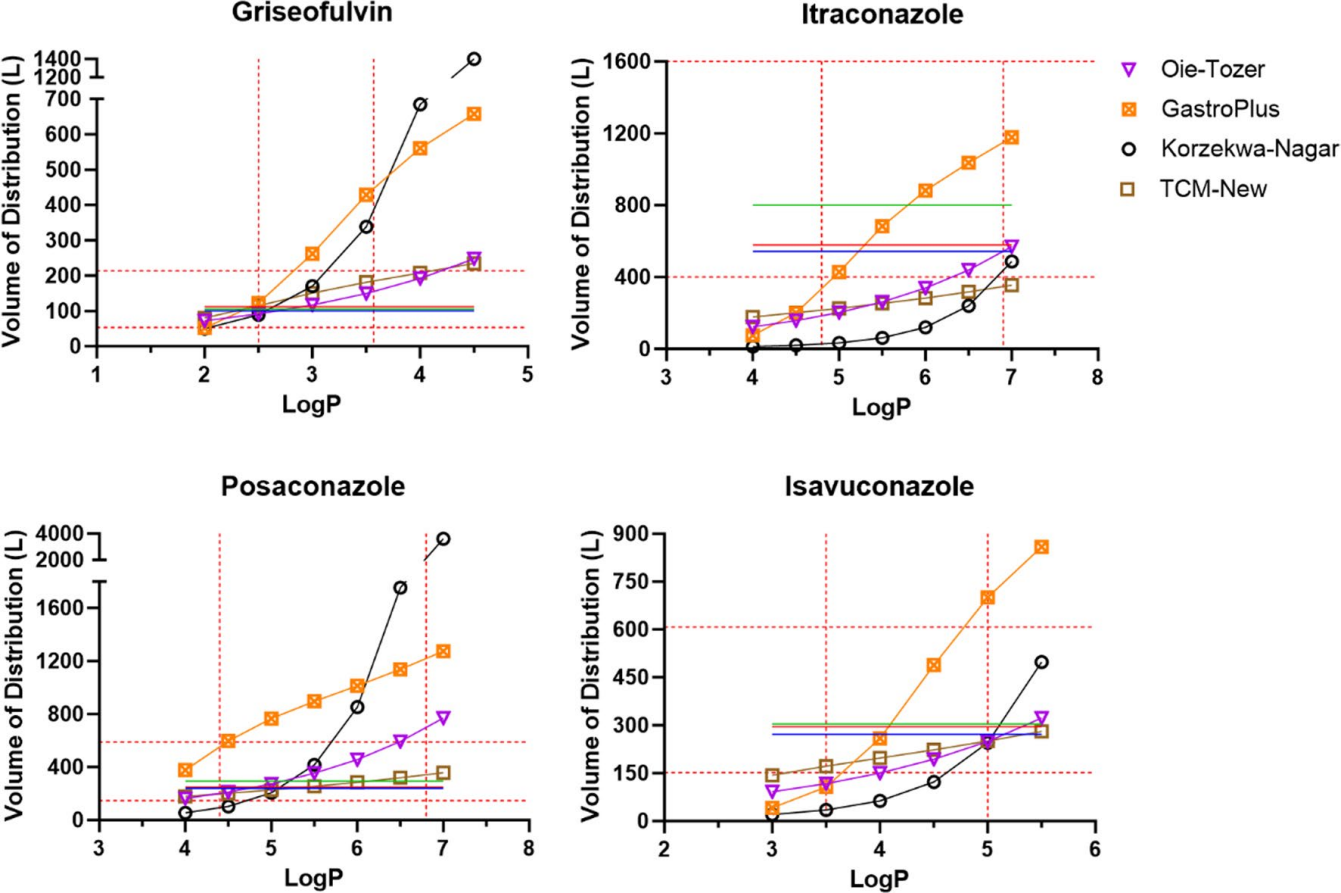
Griseofulvin, itraconazole, posaconazole, and isavuconazole VD_ss_ across a range of logP values. Profiles are volume of distribution from NCA here from plasma concentration *vs* time reported in the literature (blue circle); compartmental analysis here of plasma concentration *vs* time from the literature (red square); reported in the literature (green line); Oie-Tozer (purple diamond); GastroPlus (orange square with x); Korzekwa-Nagar (open circle); and TCM-New (open square). The lower and upper limits of reported (literature and HPLC-based) and ADMET Predictor logP values are denoted as red dash lines in the X-axis (Table II). The twofold error boundaries from the literature VD_ss_ are denoted as red dash lines in the Y-axis. Table S13 lists plotted values.

**Fig. 5:**
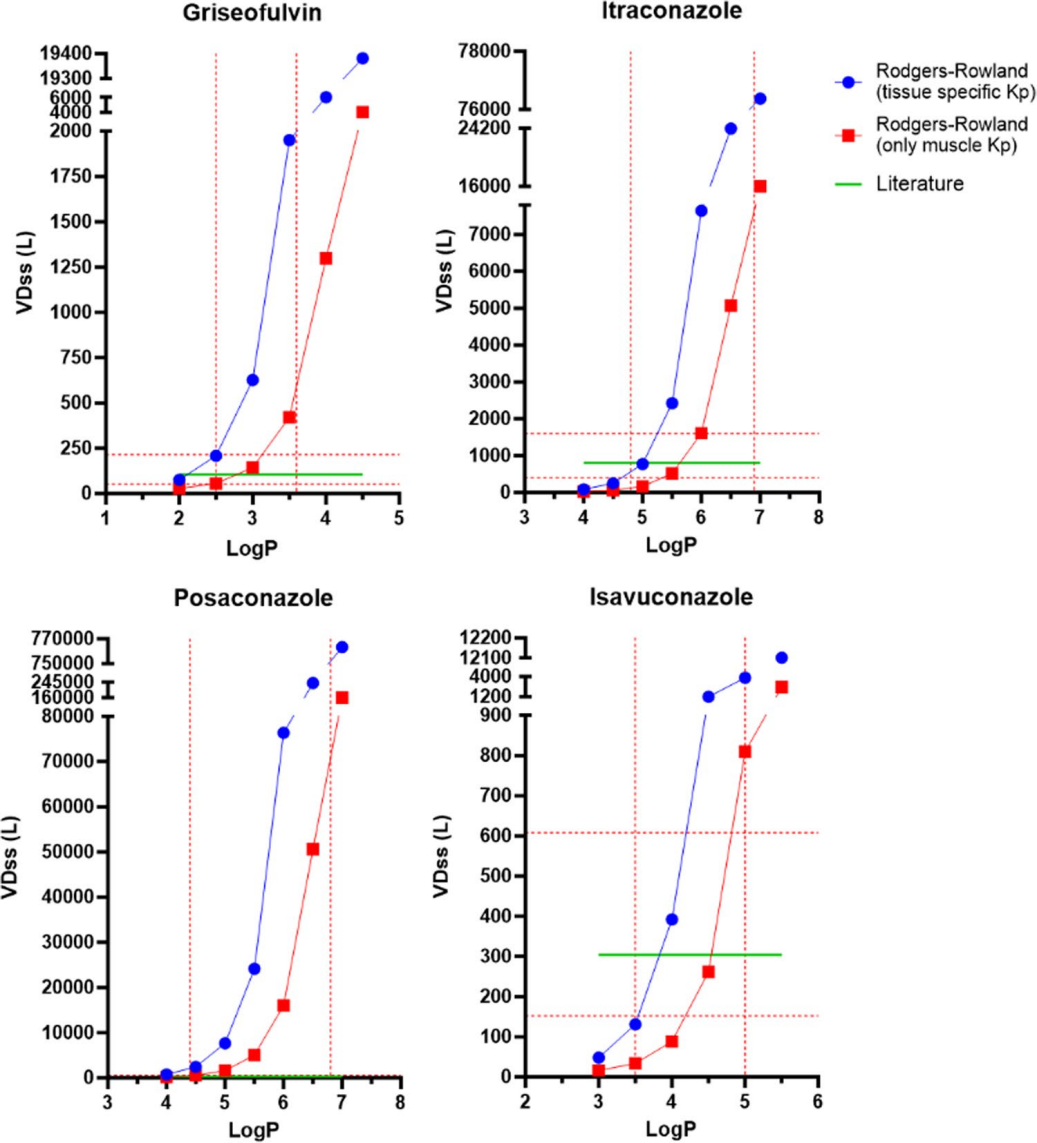
Griseofulvin, itraconazole, posaconazole, and isavuconazole VD_ss_ across a range of logP values for Rodgers-Rowland methods. Literature VD_ss_ is denoted as green line. VD_ss_ was calculated using Rodgers-Rowland equation (Eqs. 4 and 5) using tissue-specific Kp (blue circle) and only muscle Kp (red square). VD_ss_ results here are much larger than those in Fig. 4. The lower and upper limits of reported (literature and HPLC-based) and ADMET Predictor logP values are denoted as red dash lines in the X-axis (Table II). The twofold error boundaries from the literature VD_ss_ are denoted as red dash lines in the Y-axis. Table S13 lists plotted values.

Figure 4 shows VD_ss_ from Oie-Tozer, GastroPlus, Korzekwa-Nagar, and TCM-New, along with NCA, compartmental analysis, and literature values. Since Rodgers-Rowland VD_ss_ are very large, they are plotted separately in Fig. 5, along with literature values.

Human VD_ss_ from the literature and NCA and compartmental analysis are not logP-dependent; they are represented by straight lines in Figs. 4 and 5. The twofold error boundaries from the literature VD_ss_ are shown as red dashed lines on the Y-axis. Overall, for each drug, Oie-Tozer, Rodgers-Rowland (tissue-specific and muscle-only Kp), GastroPlus VD_ss_, and Korzekwa-Nagar predictions increased with higher logP values.

#### Oie-Tozer

In Fig. 4, Oie-Tozer VD_ss_ predictions for all four drugs increased slightly with logP increments. For griseofulvin, VD_ss_ predictions were within twofold error of observed VD_ss_ for most logP values (i.e., predicted VD_ss_ values were within the red dashed lines on the Y-axis). On the other hand, Oie-Tozer underpredicted itraconazole VD_ss_ for most of the logP range. Posaconazole VD_ss_ were mostly within twofold error. Isavuconazole VD_ss_ values were underpredicted at lower logP values, but within twofold error for logP > 4. Compared to the other methods, Oie-Tozer VD_ss_ did not increase substantially at high logP values. Even at higher logP values, Oie-Tozer VD_ss_ predictions were within twofold error.

#### Rodgers-Rowland (Tissue-Specific Kp)

In Fig. 5, there was an underprediction of itraconazole and isavuconazole at lower logP values. However, the steep increase in VD_ss_ *versus* logP from Rodgers-Rowland (tissue-specific Kp) showed that this approach was not suitable for highly lipophilic compounds. VD_ss_ was overpredicted when logP was greater or equal to 3, 5.5, 4, and 4.5 for griseofulvin, itraconazole, posaconazole, and isavuconazole, respectively. As logP increased, VD_ss_ predictions were astonishingly high. For example, posaconazole VD_ss_ prediction at logP = 4.5 was eightfold higher than the observed human VD_ss_; but this overprediction increases to 80-fold when logP was 5.5.

VD_ss_ overprediction from Rodgers-Rowland (tissue-specific Kp) when drug logP > 3 has been discussed previously, including by Rodgers and Rowland [10, 12, 16, 32]. A previous study suggested that this method was not suitable for drugs with logP greater than 4 [12]. The current analysis provides a more detailed and quantified analysis of the limitation of VD_ss_ prediction of four lipophilic drugs. Despite the limited number of drugs here in this study, it seems inappropriate to apply the Rodgers-Rowland method (tissue-specific Kp or only-muscle Kp) to drugs with logP > 3.

#### Rodgers-Rowland (Only Muscle Kp)

In Fig. 5, the slope from only using muscle Kp was not as steep as the tissue-specific Kp method. However, higher logP still yielded VD_ss_ values that were much higher than observed VD_ss_. VD_ss_ was overpredicted when logP was greater or equal to 3.5, 6.5, 5, and 5 for griseofulvin, itraconazole, posaconazole, and isavuconazole, respectively. Despite underpredicting VD_ss_ for itraconazole for lower logP, VD_ss_ was overpredicted by 20 times at logP = 7. Similar to itraconazole, isavuconazole VD_ss_ was underpredicted for most logP values, but showed VD_ss_ overprediction by eightfold at logP = 5.5. Even though muscle Kp is not as sensitive to logP values as adipose tissue Kp (compare Fig. 5 to Fig. 3), and yielded underprediction for logP values less than 4.5 in general, muscle Kp still increased excessively with an increase in logP value. For example, VD_ss_ prediction was over 100-fold higher than observed VD_ss_ when logP was around 6.5 for posaconazole. Therefore, even the Rodgers-Rowland method that uses only muscle Kp for all tissues is not recommended for highly lipophilic drugs, because of the markedly overpredicted VD_ss_ values at high logP.

#### GastroPlus

In Fig. 4, the GastroPlus approach to calculate Kp performed better than the Rodgers-Rowland method in Fig. 5, as the VD_ss_ predictions were closer to the observed human VD_ss_ values. The improvement here with neutral and weakly basic drugs (pKa < 7) stems from the adjustment of fup in the Kp predictions. The adjusted fup used for each logP estimate is presented in Table S8.

Despite this relative improvement, GastroPlus overpredicted VD_ss_ for griseofulvin, posaconazole, and isavuconazole at logP values starting at 3, 5, and 5 respectively. Like Rodgers-Rowland, GastroPlus VD_ss_ predictions increased markedly at higher logP for all model drugs, but the fup correction kept predictions within at most a sixfold overprediction.

Itraconazole was the only drug with VD_ss_ predictions within twofold from the observed values for most of the logP values tested. In addition, posaconazole VD_ss_ overpredictions were around fourfold larger than the observed human VD_ss_. These overpredictions are much smaller than the overpredictions from any of the Rodgers-Rowland methods, which were over 100-fold too high.

#### Korzekwa-Nagar

In Fig. 4, Korzekwa-Nagar VD_ss_ predictions increased with logP increments across all four drugs. Griseofulvin and posaconazole showed the steepest changes in VD_ss_. For these drugs, this method showed predictions within twofold error for lowest logP values in the range tested. As logP values increased, VD_ss_ predictions increased substantially for griseofulvin and posaconazole. Meanwhile, the Korzekwa-Nagar method underpredicted VD_ss_ for itraconazole and isavuconazole for most of the range of logP values tested. The underprediction of itraconazole VD_ss_ can be explained by this drug’s low fup as Korzekwa-Nagar is known to be sensitive to low fup values, and the development of this model excluded drugs with fup < 0.005 [20]. Itraconazole and isavuconazole showed VD_ss_ values that were within twofold error when logP was higher than 6.5 and 4.5, respectively. However, the VD_ss_ values increase meaningfully and VD_ss_ predictions were not within the twofold error range for many of the logP values.

#### TCM-New

In Fig. 4, the TCM-New method showed the lowest increase in VD_ss_ predictions with an increase in logP values. For griseofulvin, posaconazole, and isavuconazole, the VD_ss_ predictions were mostly within the twofold error range across the logP values tested. On the other hand, the TCM-new method underpredicted itraconazole VD_ss_ even at high logP values (logP > 5). Like the Oie-Tozer method, the TCM-New method was not overly sensitive to logP and did not increase substantially for increasingly larger logP values.

### Sensitivity Analysis: Overall Observations

For each model drug, VD_ss_ predictions using TCM-New and Oie-Tozer methods increased about 3- and fourfold across the logP range, respectively. GastroPlus VD_ss_ predictions increased about 15-fold for griseofulvin, itraconazole, and isavuconazole; surprisingly, posaconazole VD_ss_ predictions increased only about threefold.

The Korzekwa-Nagar method increased VD_ss_ predictions by 25–35-fold for griseofulvin and isavuconazole, 35-fold for itraconazole, and 66-fold for posaconazole. Meanwhile, both Rodgers-Rowland methods increased VD_ss_ predictions across the logP range by approximately 150-fold or more for all four drugs. Of the four drugs, Rodgers-Rowland overpredicted posaconazole most dramatically, as posaconazole has both relatively high logP and high fup for a lipophilic drug (i.e., fup = 0.02). GastroPlus corrects fup, such that Kp and VD_ss_ were not as overpredicted as Rodgers-Rowland.

The TCM-New and Oie-Tozer were less sensitive than Rodgers-Rowland, GastroPlus, and Korzekwa-Nagar to an increase in logP for all model drugs. Oie-Tozer underpredicted VD_ss_ values for itraconazole when logP was between 4 and 6, and TCM-New underpredicted itraconazole VD_ss_ for the logP range of 4 to 7.

### Prediction Error Per Source of logP

Predicted VD_ss_ for specific logP values (Table II) are listed in Table S14. AFE and AAFE of the six methods, grouped by the source of logP values, is presented in Table V; fold error values are presented in Table S15. Across the four drugs, rank-order log P values were: ADMET Predictor logP < literature logP < HPLC-based logP. For isavuconazole, ADMET Predictor and literature logP were virtually the same.

**Table V:** AAFE and AFE Values for VD_ss_ for Each Method Across Different Sources of logP Values. LogP Sources were ADMET Predictor logP, Literature logP, or HPLC-Based logP. Fold Error Values are Listed in Table S15. The Reported Human VD_ss_ Values for Griseofulvin, Itraconazole, Posaconazole, and Isavuconazole were 107, 800, 294, and 304 L, Respectively

		Oie-Tozer		Rodgers-Roland (tissue-specific Kp)		Rodgers-Roland (muscle only Kp)		GastroPlus		Korzekwa-Nagar		TCM-New	
ADMET Predictor logP		AFE	AAFE	AFE	AAFE	AFE	AAFE	AFE	AAFE	AFE	AAFE	AFE	AAFE
Griseofulvin	2.511	0.49	2.03	1.54	1.54	0.36	2.77	0.82	1.22	0.21	4.81	0.59	1.71
Itraconazole	4.893												
Posaconazole	4.405												
Isavuconazole	3.619												
Literature logP		AFE	AAFE	AFE	AAFE	AFE	AAFE	AFE	AAFE	AFE	AAFE	AFE	AAFE
Griseofulvin	3.53	0.69	1.45	7.06	7.06	1.56	1.56	1.45	1.45	0.46	2.17	0.72	1.39
Itraconazole	5.66												
Posaconazole	5.36												
Isavuconazole	3.56												
HPLC-based logP		AFE	AAFE	AFE	AAFE	AFE	AAFE	AFE	AAFE	AFE	AAFE	AFE	AAFE
Griseofulvin	3.566	1.15	1.15	69.17	69.17	14.62	14.62	2.71	2.71	1.81	1.81	0.91	1.10
Itraconazole	6.888												
Posaconazole	6.716												
Isavuconazole	4.934												

Oie-Tozer successfully predicted VD_ss_ within twofold using literature and HPLC-based logP values in about half of the cases. AFE and AAFE were slightly outside the twofold error range at 0.49 and 2.03, respectively for ADMET Predictor logP values. Oie-Tozer underpredicted VD_ss_ for itraconazole and isavuconazole, leading to an AFE and AAFE outside the twofold range. Oie-Tozer predictions were more accurate as logP increased (AAFE = 1.15 for HPLC-based, 1.45 for literature, 2.03 for ADMET Predictor sources).

In contrast to Oie-Tozer, Rodgers-Rowland using tissue-specific Kp results were less accurate as logP values increased. The AFE (and AAFE) were 1.54, 7.06, and 69.17 for ADMET Predictor, literature, and HPLC-based logP, respectively. Therefore, the apparent best data source for Rodgers-Rowland (tissue specific Kp) was ADMET Predictor, which again provided the lowest logP values. As logP increased, the Kp and VD_ss_ predictions from Rodgers-Rowland increased dramatically and over-predicted.

Rodgers-Rowland using only muscle Kp on average underpredicted VD_ss_ when logP values from ADMET predictor were used (AFE = 0.36 and AAFE = 2.77) and overpredicted when HPLC-based logP valued were used (AFE = 14.62 and AAFE = 14.62). This method appeared successful in predicting VD_ss_ when literature logP was used (AFE = 1.56 and AAFE = 1.56). However, when examining specific fold errors for logP values from the literature, Rodgers-Rowland method (only muscle Kp) substantially underpredicted isavuconazole VD_ss_ (fold error = 0.16) and overpredicted posaconazole VD_ss_ (fold error = 12.50), both of which are undesirable, even though the AFE and AAFE, indicated success on average.

Both Rodgers-Rowland methods were very sensitive to drug fup. Griseofulvin and isavuconazole have similar literature logP values (3.53 and 3.56, respectively), but very different fup values (0.16 and 0.1, respectively). Griseofulvin is a neutral drug, while isavuconazole is a weak base (pKa = 3.28), but there are minor differences in X, Y, and [PR]_T_/[PR]_P_ terms in the Kp equation (Eq. 3). Therefore, fup had a large impact on VD_ss_ predictions of highly lipophilic drugs from both Rodgers-Rowland methods.

GastroPlus aims for an adjustment in fup, relative to Rodgers-Rowland. This adjustment resulted in predictions that were within the twofold error prediction error for logP values from ADMET Predictor (AFE = 0.82 and AAFE = 1.22) and literature logP values (AFE and AAFE = 1.45). Predictions using the higher valued HPLC-based logP were above the twofold error boundary, but only modestly too high (i.e., AFE and AAFE = 2.71).

The Korzekwa-Nagar method successfully predicted VD_ss_ with HPLC-based logP values (AFE = 1.81 and AAFE = 1.81), but underpredicted with ADMET Predictor (AFE = 0.21 and AAFE = 4.81) and literature logP values (AFE = 0.46 and AAFE = 2.17). Itraconazole VD_ss_ values were the source of the underperformance of the Korzekwa-Nagar method. Drugs with fup < 0.005 such as itraconazole (i.e., fup = 0.002) were not included in the Korzekwa-Nagar method development [8, 20]. Of note, ADMET Predictor was exclusively used to calculate BPR, as only itraconazole’s measured BPR (i.e., 0.58) was available in the literature [13]. Using this BPR value for itraconazole, a slightly smaller VD_ss_ by 13% was predicted, which had no impact on the relative performance of the six methods.

The TCM-New method successfully predicted VD_ss_ within twofold error for all sources of logP values. In addition, the best AFE and AAFE was when using HPLC-based logP values (AFE = 0.88 and AAFE = 1.13).

Overall, given ADMET Predictor logP < literature logP < HPLC-based logP, GastroPlus was the best method for lower logP values (i.e., ADMET Predictor logP), and TCM-New was the best method for intermediate and higher logP values (i.e., literature and HPLC-based logP) (Table V). These findings show that, overall, Oie-Tozer, GastroPlus, Korzekwa-Nagar, and TCM-New provided more accurate VD_ss_ prediction than Rodgers-Rowland methods. TCM-New was the only method that was accurate regardless of logP value source among the six tested methods. TCM-New has been shown to be an accurate prediction method for highly lipophilic compounds [13].

### Prediction Errors Per Specific Drug

**Fig. 6:**
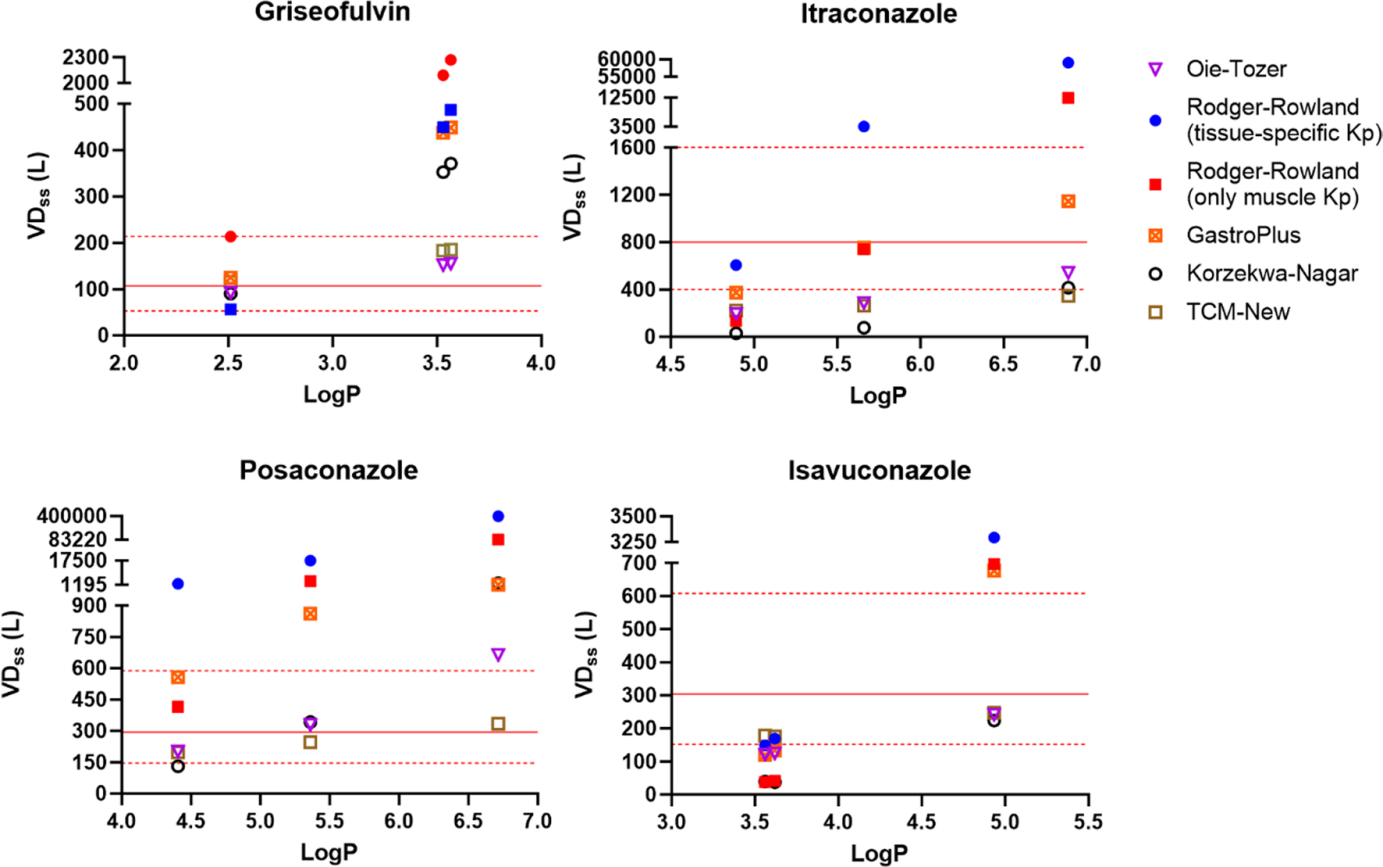
Griseofulvin, itraconazole, posaconazole, and isavuconazole VD_ss_ at specific reported logP. Profiles are VD_ss_ predicted from Oie-Tozer (purple diamond); Rodgers-Rowland (tissue-specific Kp) (blue circle); Rodgers-Rowland (only muscle Kp) (red square); GastroPlus (orange square with x); Korzekwa-Nagar (open circle); and TCM-New (open square). The literature VD_ss_ from human clinical trials is denoted by red line and twofold error is denoted by red dashed line. Plotted values are listed in Table S14.

An analysis was also conducted with a focus on each individual drug, while considering the range of logP values of each drug. This analysis recognizes that logP for any drug has uncertainty, along with a potentially steep dependence of VD_ss_ on logP in the high logP range. The results of prediction error analysis on each individual drug and their reported logP values are presented in Fig. 6, Table VI, and Table S16. In Fig. 6, the red solid line and the dashed red lines represent the VD_ss_ reported in the literature from human clinical studies and the boundaries for the twofold error prediction, respectively.

**Table VI:** AAFE and AFE Values for VD_ss_ for each Method Across Griseofulvin, Itraconazole, Posaconazole, and Isavuconazole. Fold Error Values are Listed in Table S16. The Reported Human VD_ss_ Values for Griseofulvin, Itraconazole, Posaconazole, and Isavuconazole were 107, 800, 294, and 304 L, Respectively

		Oie-Tozer		Rodgers-Roland (tissue-specific Kp)		Rodgers-Roland (muscle only Kp)		GastroPlus		Korzekwa-Nagar		TCM-New	
Drug	LogP	AFE	AAFE	AFE	AAFE	AFE	AAFE	AFE	AAFE	AFE	AAFE	AFE	AAFE
Griseofulvin	2.511	1.21	1.21	9.38	9.38	6.35	6.35	2.71	2.71	2.13	2.13	1.47	1.47
	3.53												
	3.566												
Drug	LogP	AFE	AAFE	AFE	AAFE	AFE	AAFE	AFE	AAFE	AFE	AAFE	AFE	AAFE
Itraconazole	4.893	0.39	2.58	6.25	6.25	1.33	1.33	0.86	1.17	0.12	8.11	0.34	2.94
	5.66												
	6.888												
Drug	LogP	AFE	AAFE	AFE	AAFE	AFE	AAFE	AFE	AAFE	AFE	AAFE	AFE	AAFE
Posaconazole	4.41	1.20	1.20	81.08	81.08	17.10	17.10	2.83	2.83	1.62	1.62	0.86	1.16
	5.36												
	6.72												
Drug	LogP	AFE	AAFE	AFE	AAFE	AFE	AAFE	AFE	AAFE	AFE	AAFE	AFE	AAFE
Isavuconazole	3.619	0.51	1.98	1.43	1.43	0.34	2.96	0.73	1.37	0.23	4.38	0.65	1.54
	3.56												
	4.934												

For griseofulvin, VD_ss_ predictions using Oie-Tozer and TCM-New were the most accurate when considering all reported logP values. At lower logP (logP = 2.5), all VD_ss_ prediction methods were acceptable. As logP values for griseofulvin increased, both Rodgers-Rowland methods overpredicted VD_ss_ by more than fivefold. The GastroPlus and Korzekwa-Nagar methods also overpredicted VD_ss_, but to a lesser degree.

For itraconazole, at lower logP values (logP < 5), all methods underpredicted VD_ss_ by more than twofold, except Rodgers-Rowland (tissue specific Kp). As logP values increased, Rodgers-Rowland (only muscle Kp) and GastroPlus yielded the most accurate predictions. As logP values further increased, the Oie-Tozer, GastroPlus, Korzekwa-Nagar, and TCM-New methods provided good predictions within twofold error. However, both Oie-Tozer, Korzekwa-Nagar, and TCM-New methods underpredicted itraconazole VD_ss_ for most of the logP values. Itraconazole would not be a good candidate for the Korzekwa-Nagar method due to its low fup (i.e. fup = 0.002). Despite predicting itraconazole VD_ss_ within twofold error, Rodgers-Rowland (only muscle Kp) was within the twofold error because of underpredicted predictions at logP = 4.893 (fold error = 0.17) and overpredicted (fold error = 15.47) at logP = 6.888. Therefore, the best prediction method for itraconazole was highly dependent on the logP value chosen as no single prediction method yielded predictions for all three logP values within the twofold error criteria. Overall, GastroPlus method yields the best predictions across the range of itraconazole logP.

For posaconazole, at lower logP values, most methods were acceptable except for Rodgers-Rowland (tissue-specific Kp), which overpredicted VD_ss_. As logP values increased, both Rodgers-Rowland and GastroPlus methods overpredicted VD_ss_. TCM-New, Oie-Tozer and Korzekwa-Nagar had acceptable AFE and AAFE. However, the Korzekwa-Nagar method, and the Oie-Tozer to a less extent, had underpredictions and overpredictions that balanced out. Therefore, the best prediction method for posaconazole was TCM-New.

Isavuconazole VD_ss_ was underpredicted by all methods at logP values less than 4 with the exception of Rodgers-Rowland (tissue-specific Kp) and TCM-New. At higher logP (logP = 4.934), Korzekwa-Nagar, Oie-Tozer, and TCM-New VD_ss_ prediction was within the twofold error criteria, although still underpredicted VD_ss_, while all others overpredicted by more than twofold. Despite showing acceptable AFE and AAFE, Oie-Tozer, both Rodger-Rowland methods, GastroPlus, and Korzekwa-Nagar underpredicted or overpredicted VD_ss_ depending on the logP value used. Among the six tested methods, TCM-New was the only method that successfully predicted VD_ss_ for all logP values of isavuconazole.

### Prediction Errors of Combined Data

When combining all predicted VD_ss_ from the three logP sources for the four drugs, TCM-New was the most accurate method (AFE = 0.76, AAFE = 1.32). Despite having the same AFE and AAFE as the TCM-New method, the Oie-Tozer method had four underpredictions and one overprediction outside the twofold error while the TCM-New method had three underpredictions outside the twofold error (Table S15). Therefore, the TCM-New method was determined to perform better than the Oie-Tozer method.

Other methods such as GastroPlus (AFE = 1.48, AAFE = 1.48) and Korzekwa-Nagar (AFE = 0.56, AAFE = 1.79) also showed acceptable prediction errors. These findings are in accordance with previous research has shown that the Oie-Tozer to be a slightly better prediction method than GastroPlus for human VD_ss_ estimation [43]. Results here are in accordance with the literature as TCM-New has been shown to be more accurate than GastroPlus and Rodgers-Rowland (tissue-specific Kp), and specially for highly lipophilic (logP > 3) [13].

As expected, Rodgers-Rowland (tissue-specific Kp) overpredicted VD_ss_ for all four model drugs (AFE = AAFE = 9.09). Rodgers-Rowland method (only muscle Kp) also overpredicted VD_ss_ overall, but to a less extent (AFE = AAFE = 2.02). Rodger-Rowland (tissue-specific Kp) is known to overestimate VD_ss_ of highly lipophilic drugs. One of the reasons for this failure is because of the errors involved in measuring fup of lipophilic drugs [44]. The GastroPlus method attempted to overcome the Rodger-Rowland VD_ss_ overprediction by adjusting fup to calculate Kp. This strategy worked to some extent. GastroPlus overpredicted VD_ss_ to a less extent than both Rodgers-Rowland method, but still overpredicted more values than the Korzekwa-Nagar method. One of the Korzekwa-Nagar method limitations is that the method was not designed for drugs with fup > 0.005. Many highly lipophilic drugs also have very low fup, rendering this method not a good choice for these types of compounds, per itraconazole results here.

The TCM-New method offered a new approach to overcome the VD_ss_ overprediction by removing fup from the VD_ss_ model and adding BPR instead. This approach has shown to be more successful than the Rodgers-Rowland and GastroPlus methods to predict VD_ss_ of a set of 202 compounds [13]. Here, TCM-New method showed to be the best method for VD_ss_ prediction of highly lipophilic drugs, and results point towards TCM-New as advantageous in using BPR for drug partitioning into tissues and avoiding the use of fup.

## Conclusions

The goal of this study was to assess the impact of high drug logP on VD_ss_ predictions, including intermediate fut and Kp values, from six methods. This goal was motivated by the uncertainty in logP values in the literature, as reported logP values for each drug differed by at least one logP unit. This goal was also motivated by the potentially steep dependence of predicted VD_ss_ on logP (i.e., for logP > 3).

Rodgers-Rowland dramatically overpredicted VD_ss_ for lipophilic drugs, especially when logP value was particularly high. The Rodgers-Rowland methods and, to a lesser extent, GastroPlus and Korzekwa-Nagar methods were highly sensitive to logP value. The GastroPlus and Korzekwa-Nagar showed an improvement in VD_ss_ prediction errors compared to both Rodgers-Rowland methods. The GastroPlus method adjusts fup when calculating Kp and VD_ss_, while the Korzekwa-Nagar method incorporates drug orientation and physicochemical properties, besides logP, to model drug-cell membrane interactions and predict f_um_.

Overall, TCM-New was the most accurate prediction across the four drugs and three logP sources followed by the Oie-Tozer method, in part because these two methods were only modestly sensitive to logP values. Interestingly, the two most accurate predictive scenarios were TCM-New and Oie-Tozer employing HPLC-based logP values, which are higher logP values. Overall, findings suggest care in identifying and applying logP methods and values in estimating VD_ss_ in drug development and regulatory applications.

## Supplementary Information

Below is the link to the electronic supplementary material.

Supplementary file1 (DOCX 93 KB)

## Data Availability

Data can be made available upon request.

## ABBREVIATIONS

BPR: Blood-to-plasma ratio
f_i(__7.4)_: Fraction of drug ionized at pH 7.4
f_um_: Fraction unbound to microsomes
fup: Fraction of drug unbound in plasma (and/or serum)
fut: Fraction of drug unbound in tissue
Kp: Drug tissue-to-plasma partition coefficient
Kpu: Unbound tissue-to-plasma partition coefficient
LogD: Experimentally determined log of drug distribution coefficient at pH 7.4
LogP: Log of the drug partition coefficient (P)
P: Drug partition coefficient in a binary system (e.g., octanol and water)
R_e/i_: Extravascular/intravascular ratio, ratio of binding proteins in the extracellular fluid to binding proteins in plasma
V_e_: Volume of extracellular fluid
V_p_: Volume of plasma
V_r_: Volume of drug distributes to minus the extracellular fluid not considering the plasma volume
V_e_: Volume of extracellular fluid
VD_ss_: Volume of distribution at steady-state
Vu_ss_: Volume of distribution of unbound drug at steady-state
