# Supplementary material for: Relative Performance of Volume of Distribution Prediction Methods for Lipophilic Drugs with Uncertainty in LogP Value
Source: Pharm Res. 2024 May 8;41(6):1121–38. doi: 10.1007/s11095-024-03703-4 (PMC11196289; doi:10.1007/s11095-024-03703-4)
Supplement: Supplementary file 1 — Supplementary file1 (DOCX 93 KB) [file 11095_2024_3703_MOESM1_ESM.docx]

Supplementary information for

**Relative Performance of Volume of Distribution Prediction Methods for Lipophilic Drugs with Uncertainty in LogP Value**

Ana L. Coutinho^1^, Rodrigo Cristofoletti^2^, Fang Wu^3^, Abdullah Al Shoyaib^3^, Jennifer Dressman^4^, James E. Polli^1^*

^1^ Department of Pharmaceutical Sciences, University of Maryland School of Pharmacy, Baltimore, Maryland, United States of America

^2^ Department of Pharmaceutics, Center for Pharmacometrics and Systems Pharmacology, College of Pharmacy, University of Florida, Orlando, Florida, United States of America

^3^ Office of Generic Drugs, Food and Drug Administration, White Oak, Maryland, United States of America

^4^ Fraunhofer Institute of Translational Medicine and Pharmacology, Theodor-Stern-Kai 7, 60596 Frankfurt am Main, Germany

*Corresponding Author

Mailing Address: 20 Penn Street, Room 623, HSF2 Building, Baltimore, MD 21201 United States of America

Email: jpolli@rx.umaryland.edu

Telephone: 410-706-8292

Fax: 410-706-5017

The 16-digit ORCID of the author(s):

Ana Luisa Coutinho - 0000-0002-3885-3709

Rodrigo Cristofoletti – 0000-0003-2619-0343

James E. Polli - 0000-0002-5274-4314

Table S1 - Model parameters used to calculate LK_L_ in the Korzekwa-Nagar method (Equation 12).

| Model parameters - LK_L_ | Values |
| --- | --- |
| const1 | -1.36 |
| a1 | 0.63 |
| d1 | -0.55 |
| const2 | -1.55 |
| a2 | 0.53 |
| b2 | -0.12 |
| c2 | 0.21 |
| const3 | -1.56 |
| b3 | 0.26 |
| e | -0.43 |
| f | 0.77 |

Table S2 - Drug specific physicochemical parameters used to calculate LK_L_ in Korzekwa-Nagar method (Equation 12).

| **Drug** | **Dipole** | **#SO** | **#NO_2_** | **H-bond acceptors** | **H-bond donors** | **pK_a,a_** | **pK_a,b_** |
| --- | --- | --- | --- | --- | --- | --- | --- |
| Griseofulvin | 1.0254 | 0 | 0 | 6 | 0 | 14 | 1 |
| Itraconazole | 1.2653 | 0 | 0 | 9 | 0 | 14 | 4.57 |
| Posaconazole | 1.4915 | 0 | 0 | 9 | 1 | 14 | 4.67 |
| Isavuconazole | 0.8019 | 0 | 0 | 5 | 1 | 14 | 3.28 |

Table S3 - Log(fut) and fut for griseofulvin, itraconazole, posaconazole, and isavuconazole across a range of logP values found in the literature. Logfut was calculated using Equation 2. Also shown is the calculated VD_ss_ (Equation 1).

| **Griseofulvin** | | | |
| --- | --- | --- | --- |
| **LogD** | **Log(fut)** | **fut** | **VD_ss_** |
| 2 | -1.158 | 0.06951 | 72.5 |
| 2.5 | -1.273 | 0.05338 | 91.8 |
| 3 | -1.387 | 0.04099 | 117.0 |
| 3.5 | -1.502 | 0.03147 | 149.7 |
| 4 | -1.617 | 0.02417 | 192.3 |
| 4.5 | -1.731 | 0.01856 | 247.8 |
| **Itraconazole** | | | |
| **LogD** | **Log(fut)** | **fut** | **VD_ss_** |
| 4 | -3.309 | 0.00049 | 123.5 |
| 4.5 | -3.424 | 0.00038 | 158.4 |
| 5 | -3.538 | 0.00029 | 204.0 |
| 5.5 | -3.653 | 0.00022 | 263.2 |
| 6 | -3.768 | 0.00017 | 340.4 |
| 6.5 | -3.883 | 0.00013 | 441.0 |
| 7 | -3.997 | 0.00010 | 571.9 |
| **Posaconazole** | | | |
| **LogD** | **Log(fut)** | **fut** | **VD_ss_** |
| 4 | -2.419 | 0.00381 | 163.9 |
| 4.5 | -2.536 | 0.00291 | 211.8 |
| 5 | -2.650 | 0.00224 | 273.3 |
| 5.5 | -2.765 | 0.00172 | 353.3 |
| 6 | -2.880 | 0.00132 | 457.6 |
| 6.5 | -2.994 | 0.00101 | 593.5 |
| 7 | -3.109 | 0.00078 | 770.3 |
| **Isavuconazole** | | | |
| **LogD** | **Log(fut)** | **fut** | **VD_ss_** |
| 3 | -2.457 | 0.00349 | 92.1 |
| 3.5 | -2.572 | 0.00268 | 117.5 |
| 4 | -2.687 | 0.00206 | 150.5 |
| 4.5 | -2.801 | 0.00158 | 193.6 |
| 5 | -2.916 | 0.00121 | 249.6 |
| 5.5 | -3.031 | 0.00093 | 322.6 |
| 6 | -3.145 | 0.00072 | 417.7 |

Table S4 - Griseofulvin Kp_u_ and Kp using Rodgers and Rowland equation (i.e., Equation 3) for 13 tissues across different logP.

| **Griseofulvin** | |  |  |  |  |  |  |  |  |  |  |  |
| --- | --- | --- | --- | --- | --- | --- | --- | --- | --- | --- | --- | --- |
|  | **LogP = 2** | | **LogP = 2.5** | | **LogP = 3** | | **LogP = 3.5** | | **LogP = 4** | | **LogP = 4.5** | |
| **Tissue** | **Kp_u_** | **Kp** | **Kp_u_** | **Kp** | **Kp_u_** | **Kp** | **Kp_u_** | **Kp** | **Kp_u_** | **Kp** | **Kp_u_** | **Kp** |
| Adipose | 26.8 | 4.3 | 82.5 | 13.2 | 258.4 | 41.3 | 814.8 | 130.4 | 2574.3 | 411.9 | 8138.2 | 1302.1 |
| Bone | 2.5 | 0.4 | 6.3 | 1.0 | 18.4 | 2.9 | 56.8 | 9.1 | 178.1 | 28.5 | 561.8 | 89.9 |
| Brain | 4.9 | 0.8 | 13.4 | 2.2 | 40.4 | 6.5 | 125.7 | 20.1 | 395.4 | 63.3 | 1248.2 | 199.7 |
| Gut | 5.6 | 0.9 | 14.4 | 2.3 | 42.3 | 6.8 | 130.6 | 20.9 | 409.9 | 65.6 | 1293.0 | 206.9 |
| Heart | 3.0 | 0.5 | 6.6 | 1.0 | 17.7 | 2.8 | 52.8 | 8.4 | 163.9 | 26.2 | 515.3 | 82.4 |
| Kidney | 3.3 | 0.5 | 7.4 | 1.2 | 20.3 | 3.3 | 61.3 | 9.8 | 190.7 | 30.5 | 600.1 | 96.0 |
| Liver | 3.5 | 0.6 | 7.9 | 1.3 | 21.7 | 3.5 | 65.4 | 10.5 | 203.6 | 32.6 | 640.5 | 102.5 |
| Lung | 3.9 | 0.6 | 9.3 | 1.5 | 26.2 | 4.2 | 79.7 | 12.8 | 248.8 | 39.8 | 783.8 | 125.4 |
| Muscle | 2.2 | 0.4 | 4.8 | 0.8 | 13.0 | 2.1 | 39.0 | 6.2 | 121.1 | 19.4 | 380.6 | 60.9 |
| Pancreas | 5.2 | 0.8 | 14.5 | 2.3 | 43.8 | 7.0 | 136.4 | 21.8 | 429.3 | 68.7 | 1355.6 | 216.9 |
| Skin | 7.3 | 1.2 | 20.6 | 3.3 | 62.5 | 10.0 | 195.2 | 31.2 | 614.8 | 98.4 | 1941.6 | 310.7 |
| Spleen | 2.6 | 0.4 | 4.7 | 0.8 | 11.4 | 1.8 | 32.5 | 5.2 | 99.2 | 15.9 | 310.1 | 49.6 |
| Thymus | 3.1 | 0.5 | 7.3 | 1.2 | 20.5 | 3.3 | 62.4 | 10.0 | 194.7 | 31.2 | 613.3 | 98.1 |

Table S5 - Itraconazole Kp_u_ and Kp using Rodgers and Rowland equation (i.e., Equation 3) for 13 tissues across different logP.

| **Itraconazole** | |  |  |  |  |  |  |  |  |  |  |  |  | |  |
| --- | --- | --- | --- | --- | --- | --- | --- | --- | --- | --- | --- | --- | --- | --- | --- |
|  | **LogP = 4** | | **LogP = 4.5** | | **LogP = 5** | | **LogP = 5.5** | | **LogP = 6** | | **LogP = 6.5** | | **LogP = 7** | | |
| **Tissue** | **Kp_u_** | **Kp** | **Kp_u_** | **Kp** | **Kp_u_** | **Kp** | **Kp_u_** | **Kp** | **Kp_u_** | **Kp** | **Kp_u_** | **Kp** | **Kp_u_** | **Kp** | |
| Adipose | 2595.1 | 5.2 | 8151.9 | 16.3 | 25724.0 | 51.4 | 81292.0 | 162.6 | 257013.3 | 514.0 | 812692.8 | 1625.4 | 2569906.0 | 5139.8 | |
| Bone | 226.2 | 0.5 | 606.4 | 1.2 | 1808.8 | 3.6 | 5611.2 | 11.2 | 17635.3 | 35.3 | 55658.9 | 111.3 | 175900.2 | 351.8 | |
| Brain | 418.3 | 0.8 | 1269.5 | 2.5 | 3961.0 | 7.9 | 12472.3 | 24.9 | 39387.4 | 78.8 | 124500.4 | 249.0 | 393651.3 | 787.3 | |
| Gut | 486.9 | 1.0 | 1367.7 | 2.7 | 4153.1 | 8.3 | 12961.0 | 25.9 | 40814.3 | 81.6 | 128894.1 | 257.8 | 407426.9 | 814.9 | |
| Heart | 241.3 | 0.5 | 592.3 | 1.2 | 1702.3 | 3.4 | 5212.5 | 10.4 | 16312.6 | 32.6 | 51414.3 | 102.8 | 162415.5 | 324.8 | |
| Kidney | 254.8 | 0.5 | 663.9 | 1.3 | 1957.8 | 3.9 | 6049.3 | 12.1 | 18987.9 | 38.0 | 59903.2 | 119.8 | 189288.7 | 378.6 | |
| Liver | 247.4 | 0.5 | 688.0 | 1.4 | 2081.4 | 4.2 | 6487.8 | 13.0 | 20422.1 | 40.8 | 64486.1 | 129.0 | 203828.8 | 407.7 | |
| Lung | 352.2 | 0.7 | 883.8 | 1.8 | 2564.7 | 5.1 | 7880.3 | 15.8 | 24689.7 | 49.4 | 77845.8 | 155.7 | 245940.0 | 491.9 | |
| Muscle | 152.4 | 0.3 | 411.2 | 0.8 | 1229.7 | 2.5 | 3818.0 | 7.6 | 12002.8 | 24.0 | 37885.6 | 75.8 | 119734.0 | 239.5 | |
| Pancreas | 458.3 | 0.9 | 1383.3 | 2.8 | 4308.1 | 8.6 | 13557.3 | 27.1 | 42805.9 | 85.6 | 135298.1 | 270.6 | 427783.9 | 855.6 | |
| Skin | 746.7 | 1.5 | 2061.1 | 4.1 | 6217.4 | 12.4 | 19360.8 | 38.7 | 60923.8 | 121.8 | 192357.8 | 384.7 | 607988.5 | 1216.0 | |
| Spleen | 149.3 | 0.3 | 366.3 | 0.7 | 1052.4 | 2.1 | 3222.1 | 6.4 | 10083.2 | 20.2 | 31780.0 | 63.6 | 100391.3 | 200.8 | |
| Thymus | 231.5 | 0.5 | 649.4 | 1.3 | 1971.1 | 3.9 | 6150.8 | 12.3 | 19367.8 | 38.7 | 61163.9 | 122.3 | 193334.8 | 386.7 | |

Table S6 - Posaconazole Kp_u_ and Kp using Rodgers and Rowland equation (i.e., Equation 3) for 13 tissues across different logP.

| **Posaconazole** | |  |  |  |  |  |  |  |  |  |  |  |  |  |
| --- | --- | --- | --- | --- | --- | --- | --- | --- | --- | --- | --- | --- | --- | --- |
|  | **LogP = 4** | | **LogP = 4.5** | | **LogP = 5** | | **LogP = 5.5** | | **LogP = 6** | | **LogP = 6.5** | | **LogP = 7** | |
| **Tissue** | **Kp_u_** | **Kp** | **Kp_u_** | **Kp** | **Kp_u_** | **Kp** | **Kp_u_** | **Kp** | **Kp_u_** | **Kp** | **Kp_u_** | **Kp** | **Kp_u_** | **Kp** |
| Adipose | 2572.0 | 51.4 | 8126.7 | 162.5 | 25692.1 | 513.8 | 81238.8 | 1624.8 | 256893.0 | 5137.9 | 812360.1 | 16247.2 | 2568901.5 | 51378.0 |
| Bone | 181.1 | 3.6 | 561.2 | 11.2 | 1763.1 | 35.3 | 5564.1 | 111.3 | 17583.6 | 351.7 | 55592.7 | 1111.9 | 175788.0 | 3515.8 |
| Brain | 396.6 | 7.9 | 1247.4 | 24.9 | 3937.9 | 78.8 | 12445.9 | 248.9 | 39350.7 | 787.0 | 124431.2 | 2488.6 | 393479.2 | 7869.6 |
| Gut | 415.7 | 8.3 | 1296.1 | 25.9 | 4080.4 | 81.6 | 12885.0 | 257.7 | 40727.6 | 814.6 | 128773.8 | 2575.5 | 407200.1 | 8144.0 |
| Heart | 170.5 | 3.4 | 521.4 | 10.4 | 1631.0 | 32.6 | 5139.8 | 102.8 | 16235.7 | 324.7 | 51324.0 | 1026.5 | 162282.8 | 3245.7 |
| Kidney | 196.2 | 3.9 | 605.2 | 12.1 | 1898.6 | 38.0 | 5988.5 | 119.8 | 18922.1 | 378.4 | 59821.8 | 1196.4 | 189157.8 | 3783.2 |
| Liver | 208.6 | 4.2 | 649.1 | 13.0 | 2041.9 | 40.8 | 6446.7 | 128.9 | 20375.6 | 407.5 | 64422.8 | 1288.5 | 203712.2 | 4074.2 |
| Lung | 256.7 | 5.1 | 788.1 | 15.8 | 2468.4 | 49.4 | 7781.9 | 155.6 | 24584.9 | 491.7 | 77720.7 | 1554.4 | 245750.6 | 4915.0 |
| Muscle | 123.5 | 2.5 | 382.3 | 7.6 | 1200.4 | 24.0 | 3787.7 | 75.8 | 11969.4 | 239.4 | 37842.3 | 756.8 | 119659.5 | 2393.2 |
| Pancreas | 431.2 | 8.6 | 1355.7 | 27.1 | 4279.5 | 85.6 | 13525.2 | 270.5 | 42762.6 | 855.3 | 135219.4 | 2704.4 | 427593.4 | 8551.9 |
| Skin | 621.9 | 12.4 | 1935.7 | 38.7 | 6090.4 | 121.8 | 19228.8 | 384.6 | 60776.0 | 1215.5 | 192159.7 | 3843.2 | 607631.5 | 12152.6 |
| Spleen | 105.6 | 2.1 | 322.5 | 6.5 | 1008.4 | 20.2 | 3177.2 | 63.5 | 10035.7 | 200.7 | 31724.2 | 634.5 | 100309.2 | 2006.2 |
| Thymus | 197.7 | 4.0 | 615.5 | 12.3 | 1936.7 | 38.7 | 6114.7 | 122.3 | 19326.7 | 386.5 | 61106.8 | 1222.1 | 193227.1 | 3864.5 |

Table S7 - Isavuconazole Kp_u_ and Kp using Rodgers and Rowland equation (i.e., Equation 3) for 13 tissues across different logP.

| **Isavuconazole** | |  |  |  |  |  |  |  |  |  |  |  |
| --- | --- | --- | --- | --- | --- | --- | --- | --- | --- | --- | --- | --- |
|  | **LogP = 3** | | **LogP = 3.5** | | **LogP = 4** | | **LogP = 4.5** | | **LogP = 5** | | **LogP = 5.5** | |
| **Tissue** | **Kp_u_** | **Kp** | **Kp_u_** | **Kp** | **Kp_u_** | **Kp** | **Kp_u_** | **Kp** | **Kp_u_** | **Kp** | **Kp_u_** | **Kp** |
| Adipose | 262.9 | 2.6 | 819.4 | 8.2 | 2579.1 | 25.8 | 8143.7 | 81.4 | 25740.5 | 257.4 | 81386.4 | 813.9 |
| Bone | 27.9 | 0.3 | 66.0 | 0.7 | 186.4 | 1.9 | 567.2 | 5.7 | 1771.3 | 17.7 | 5579.0 | 55.8 |
| Brain | 44.9 | 0.4 | 130.2 | 1.3 | 399.7 | 4.0 | 1252.0 | 12.5 | 3947.3 | 39.5 | 12470.5 | 124.7 |
| Gut | 57.2 | 0.6 | 145.4 | 1.5 | 424.3 | 4.2 | 1306.3 | 13.1 | 4095.6 | 41.0 | 12915.9 | 129.2 |
| Heart | 32.4 | 0.3 | 67.5 | 0.7 | 178.7 | 1.8 | 530.2 | 5.3 | 1641.8 | 16.4 | 5156.9 | 51.6 |
| Kidney | 32.5 | 0.3 | 73.5 | 0.7 | 203.0 | 2.0 | 612.8 | 6.1 | 1908.4 | 19.1 | 6005.7 | 60.1 |
| Liver | 29.6 | 0.3 | 73.7 | 0.7 | 213.2 | 2.1 | 654.5 | 6.5 | 2049.9 | 20.5 | 6462.5 | 64.6 |
| Lung | 46.2 | 0.5 | 99.4 | 1.0 | 267.7 | 2.7 | 800.1 | 8.0 | 2483.4 | 24.8 | 7806.4 | 78.1 |
| Muscle | 19.1 | 0.2 | 45.0 | 0.4 | 126.9 | 1.3 | 386.1 | 3.9 | 1205.8 | 12.1 | 3797.7 | 38.0 |
| Pancreas | 49.4 | 0.5 | 142.0 | 1.4 | 434.9 | 4.3 | 1361.2 | 13.6 | 4290.1 | 42.9 | 13552.3 | 135.5 |
| Skin | 89.0 | 0.9 | 220.6 | 2.2 | 636.8 | 6.4 | 1953.0 | 19.5 | 6115.1 | 61.2 | 19276.9 | 192.8 |
| Spleen | 20.2 | 0.2 | 41.9 | 0.4 | 110.7 | 1.1 | 327.9 | 3.3 | 1015.0 | 10.2 | 3187.7 | 31.9 |
| Thymus | 27.5 | 0.3 | 69.4 | 0.7 | 201.8 | 2.0 | 620.3 | 6.2 | 1943.9 | 19.4 | 6129.3 | 61.3 |

Table S8 - Adjusted fup in GastroPlus software to calculate tissue Kp based on drug logP (i.e., Equation 9). The experimental fup for griseofulvin, itraconazole, posaconazole, and isavuconazole was 0.16, 0.002, 0.02, and 0.01, respectively.

| **LogP** | **Griseofulvin Adjusted fu_p_** | **Itraconazole Adjusted fu_p_** | **Posaconazole Adjusted fu_p_** | **Isavuconazole Adjusted fu_p_** |
| --- | --- | --- | --- | --- |
| **2** | 0.146 | ---- | ---- | ---- |
| **2.5** | 0.122 | ---- | ---- | ---- |
| **3** | 0.081 | ---- | ---- | 0.00943 |
| **3.5** | 0.039 | ---- | ---- | 0.00839 |
| **4** | 0.015 | 0.0017831 | 0.0090306 | 0.00622 |
| **4.5** | 0.005 | 0.001443 | 0.0041312 | 0.00342 |
| **5** | ---- | 0.0009022 | 0.001521 | 0.00141 |
| **5.5** | ---- | 0.000413 | 0.000508 | 0.00049 |
| **6** | ---- | 0.00015 | 0.000163 | ---- |
| **6.5** | ---- | 0.00005066 | 0.0000519 | ---- |
| **7** | ---- | 0.0000163 | 0.0000165 | ---- |

Table S9 - Griseofulvin Kp using GastroPlus software across different logP. Software employes Equations 3, 7-9 and a Perfusion Limited Model.

| **Griseofulvin** |  |  |  |  |  |  |  |
| --- | --- | --- | --- | --- | --- | --- | --- |
|  | **Tissue Volume (mL)** | **LogP = 2** | **LogP = 2.5** | **LogP = 3** | **LogP = 3.5** | **LogP = 4** | **LogP = 4.5** |
| **Tissue** |  | **Kp** | **Kp** | **Kp** | **Kp** | **Kp** | **Kp** |
| Lung | 1140.7 | 0.3 | 0.4 | 0.6 | 0.8 | 0.9 | 0.9 |
| Adipose | 22937.2 | 1.0 | 2.7 | 6.4 | 11.1 | 15.2 | 18.5 |
| Muscle | 24669.52 | 0.5 | 1.1 | 2.2 | 3.3 | 3.9 | 4.2 |
| Liver | 1565.84 | 0.9 | 1.9 | 3.6 | 5.4 | 6.4 | 6.8 |
| Gut | 0 | 0.0 | 0.0 | 0.0 | 0.0 | 0.0 | 0.0 |
| Spleen | 170.01 | 0.7 | 1.3 | 2.3 | 3.4 | 4.0 | 4.2 |
| Heart | 333.69 | 0.5 | 0.9 | 1.5 | 2.2 | 2.5 | 2.7 |
| Brain | 1492.65 | 1.1 | 2.8 | 5.6 | 8.5 | 10.2 | 10.8 |
| Kidney | 355.45 | 0.6 | 1.2 | 2.2 | 3.3 | 3.9 | 4.1 |
| Skin | 2788.93 | 0.6 | 1.4 | 2.7 | 4.0 | 4.8 | 5.1 |
| Reproductive organs | 49.2 | 0.7 | 1.3 | 2.3 | 3.3 | 3.9 | 4.2 |
| Red Marrow | 1090.76 | 1.3 | 3.0 | 6.2 | 9.3 | 11.1 | 11.9 |
| Yellow Marrow | 3031.94 | 1.0 | 2.7 | 6.4 | 11.1 | 15.2 | 18.5 |
| Rest of Body | 2606.1 | 0.6 | 1.2 | 2.3 | 3.3 | 3.9 | 4.2 |

Table S10 - Itraconazole Kp using GastroPlus software across different logP. Software employes Equations 3, 7-9 and a Perfusion Limited Model.

| **Itraconazole** |  |  |  |  |  |  |  |  |
| --- | --- | --- | --- | --- | --- | --- | --- | --- |
|  | **Tissue Volume (mL)** | **LogP = 4** | **LogP = 4.5** | **LogP = 5** | **LogP = 5.5** | **LogP = 6** | **LogP = 6.5** | **LogP = 7** |
| **Tissue** |  | **Kp** | **Kp** | **Kp** | **Kp** | **Kp** | **Kp** | **Kp** |
| Lung | 1140.7 | 0.3 | 0.4 | 0.6 | 0.8 | 0.9 | 0.9 | 1.0 |
| Adipose | 24037.02 | 1.9 | 5.4 | 12.0 | 19.8 | 26.3 | 31.6 | 36.8 |
| Muscle | 25072.53 | 0.5 | 1.2 | 2.4 | 3.4 | 4.0 | 4.2 | 4.3 |
| Liver | 1585.57 | 0.8 | 2.0 | 3.9 | 5.6 | 6.5 | 6.8 | 6.9 |
| Gut | 0 | 0.0 | 0.0 | 0.0 | 0.0 | 0.0 | 0.0 | 0.0 |
| Spleen | 170.01 | 0.6 | 1.3 | 2.4 | 3.4 | 4.0 | 4.2 | 4.3 |
| Heart | 338.09 | 0.4 | 0.9 | 1.6 | 2.2 | 2.6 | 2.7 | 2.7 |
| Brain | 1492.65 | 1.3 | 3.1 | 6.2 | 8.9 | 10.3 | 10.9 | 11.1 |
| Kidney | 359.49 | 0.6 | 1.3 | 2.4 | 3.4 | 3.9 | 4.1 | 4.2 |
| Skin | 2823.67 | 0.8 | 1.7 | 3.0 | 4.3 | 4.9 | 5.2 | 5.3 |
| Reproductive organs | 50.35 | 0.6 | 1.3 | 2.4 | 3.4 | 3.9 | 4.1 | 4.2 |
| Red Marrow | 1103.51 | 1.5 | 3.5 | 6.8 | 9.8 | 11.4 | 12.0 | 12.2 |
| Yellow Marrow | 3067.36 | 1.9 | 5.4 | 12.0 | 19.8 | 26.3 | 31.6 | 36.8 |
| Rest of Body | 2666.79 | 0.6 | 1.3 | 2.4 | 3.5 | 4.0 | 4.2 | 4.3 |

Table S11 - Posaconazole Kp using GastroPlus software across different logP. Software employes Equations 3, 7-9 and a Perfusion Limited Model.

| **Posaconazole** |  |  |  |  |  |  |  |  |
| --- | --- | --- | --- | --- | --- | --- | --- | --- |
|  | **Tissue Volume (mL)** | **LogP = 4** | **LogP = 4.5** | **LogP = 5** | **LogP = 5.5** | **LogP = 6** | **LogP = 6.5** | **LogP = 7** |
| **Tissue** |  | **Kp** | **Kp** | **Kp** | **Kp** | **Kp** | **Kp** | **Kp** |
| Lung | 1140.7 | 0.6 | 0.8 | 0.9 | 0.9 | 1.0 | 1.0 | 1.0 |
| Adipose | 26176.6 | 9.2 | 15.2 | 20.2 | 24.3 | 28.2 | 32.4 | 37.0 |
| Muscle | 25852.52 | 2.4 | 3.4 | 4.0 | 4.2 | 4.3 | 4.3 | 4.3 |
| Liver | 1623.32 | 3.9 | 5.6 | 6.5 | 6.8 | 6.9 | 7.0 | 7.0 |
| ACAT Gut | 0 | 0.0 | 0.0 | 0.0 | 0.0 | 0.0 | 0.0 | 0.0 |
| Spleen | 170.01 | 2.4 | 3.4 | 4.0 | 4.2 | 4.3 | 4.3 | 4.3 |
| Heart | 346.8 | 1.6 | 2.2 | 2.6 | 2.7 | 2.7 | 2.8 | 2.8 |
| Brain | 1492.65 | 6.2 | 8.9 | 10.3 | 10.9 | 11.1 | 11.2 | 11.2 |
| Kidney | 367.17 | 2.4 | 3.4 | 3.9 | 4.1 | 4.2 | 4.2 | 4.2 |
| Skin | 2890.09 | 3.0 | 4.3 | 4.9 | 5.2 | 5.3 | 5.3 | 5.3 |
| Reproductive organs | 52.57 | 2.4 | 3.4 | 3.9 | 4.1 | 4.2 | 4.2 | 4.2 |
| Red Marrow | 1128.24 | 6.8 | 9.8 | 11.3 | 12.0 | 12.2 | 12.2 | 12.3 |
| Yellow Marrow | 3136.13 | 9.2 | 15.2 | 20.2 | 24.3 | 28.2 | 32.4 | 37.0 |
| Rest of Body | 2784.6 | 2.4 | 3.5 | 4.0 | 4.2 | 4.3 | 4.3 | 4.3 |

Table S12 - Isavuconazole Kp using GastroPlus software across different logP. Software employes Equations 3, 7-9 and a Perfusion Limited Model.

| **Isavuconazole** |  |  |  |  |  |  |  |
| --- | --- | --- | --- | --- | --- | --- | --- |
|  | **Tissue Volume (mL)** | **LogP = 3** | **LogP = 3.5** | **LogP = 4** | **LogP = 4.5** | **LogP = 5** | **LogP = 5.5** |
| **Tissue** |  | **Kp** | **Kp** | **Kp** | **Kp** | **Kp** | **Kp** |
| Lung | 1140.7 | 0.3 | 0.3 | 0.5 | 0.7 | 0.9 | 0.9 |
| Adipose | 25592.49 | 0.8 | 2.4 | 6.4 | 12.6 | 18.8 | 23.7 |
| Muscle | 25640.11 | 0.3 | 0.8 | 1.7 | 2.8 | 3.7 | 4.1 |
| Liver | 1613.09 | 0.5 | 1.2 | 2.7 | 4.6 | 6.0 | 6.7 |
| ACAT Gut | 0 | 0.0 | 0.0 | 0.0 | 0.0 | 0.0 | 0.0 |
| Spleen | 170.01 | 0.4 | 0.8 | 1.7 | 2.9 | 3.7 | 4.1 |
| Heart | 344.4 | 0.3 | 0.6 | 1.1 | 1.9 | 2.4 | 2.6 |
| Brain | 1492.65 | 0.7 | 1.9 | 4.3 | 7.4 | 9.6 | 10.6 |
| Kidney | 365.09 | 0.4 | 0.8 | 1.7 | 2.8 | 3.7 | 4.0 |
| Skin | 2872.11 | 0.6 | 1.1 | 2.2 | 3.6 | 4.6 | 5.1 |
| Reproductive organs | 51.97 | 0.4 | 0.8 | 1.7 | 2.8 | 3.7 | 4.0 |
| Red Marrow | 1121.5 | 0.9 | 2.1 | 4.7 | 8.1 | 10.6 | 11.7 |
| Yellow Marrow | 3117.37 | 0.8 | 2.4 | 6.4 | 12.6 | 18.8 | 23.7 |
| Rest of Body | 2752.47 | 0.4 | 0.8 | 1.7 | 2.9 | 3.7 | 4.1 |

Table S13 – Griseofulvin, itraconazole, posaconazole, and isavuconazole VD_ss_ in liters using the Oie-Tozer, Rodgers-Rowland (tissue-specific Kp and muscle Kp), GastroPlus, Korzekwa-Nagar, and TCM-New methods across a range of logP values.

| **Drug** | **LogP** | **Oie-Tozer** | **Rodgers-Rowland (tissue-specific Kp)** | **Rodgers-Rowland**  **(only-muscle Kp)** | **GastroPlus** | **Korzekwa-Nagar** | **TCM-New** |
| --- | --- | --- | --- | --- | --- | --- | --- |
| Griseofulvin | 2 | 72.5 | 76.0 | 27.5 | 51.6 | 50.0 | 80.5 |
|  | 2.5 | 91.8 | 208.4 | 55.2 | 122.4 | 89.3 | 114.7 |
|  | 3 | 117 | 627.1 | 143.0 | 261.7 | 170.5 | 150.8 |
|  | 3.5 | 149.7 | 1951.4 | 420.7 | 428.6 | 338.2 | 181.1 |
|  | 4 | 192.3 | 6138.8 | 1298.7 | 561.0 | 684.6 | 208.0 |
|  | 4.5 | 247.8 | 19380.9 | 4075.1 | 657.9 | 1400.1 | 234.8 |
| Itraconazole | 4 | 123.2 | 86.0 | 23.9 | 76.611 | 13.8 | 178.6 |
|  | 4.5 | 158 | 251.1 | 58.5 | 201.0 | 20.4 | 201.6 |
|  | 5 | 203.4 | 773.3 | 168.0 | 429.1 | 34.0 | 226.3 |
|  | 5.5 | 262.5 | 2424.6 | 514.1 | 683.2 | 62.1 | 253.5 |
|  | 6 | 339.5 | 7646.4 | 1608.7 | 882.4 | 120.2 | 283.9 |
|  | 6.5 | 439.8 | 24159.2 | 5070.2 | 1036.5 | 240.1 | 317.8 |
|  | 7 | 570.4 | 76377.4 | 16016.5 | 1178.2 | 487.8 | 355.9 |
| Posaconazole | 4 | 163.9 | 773.7 | 168.7 | 377.4 | 54.7 | 179.4 |
|  | 4.5 | 211.8 | 2424.4 | 514.7 | 596.6 | 104.2 | 202.5 |
|  | 5 | 273.3 | 7644.2 | 1608.9 | 766.4 | 206.4 | 227.3 |
|  | 5.5 | 353.3 | 24150.7 | 5069.1 | 896.1 | 417.5 | 254.6 |
|  | 6 | 457.6 | 76349 | 16011.2 | 1014.3 | 853.4 | 285.1 |
|  | 6.5 | 593.5 | 241414.3 | 50613 | 1137.6 | 1753.8 | 319.2 |
|  | 7 | 770.3 | 763396.6 | 160033.7 | 1274.1 | 3613.4 | 357.4 |
| Isavuconazole | 3 | 92.1 | 48.2 | 16.2 | 41.4 | 20.9 | 143.4 |
|  | 3.5 | 117.5 | 130.9 | 33.6 | 106.7 | 34.8 | 172.2 |
|  | 4 | 150.5 | 392.3 | 88.4 | 259.3 | 63.6 | 197.7 |
|  | 4.5 | 193.6 | 1219.1 | 261.7 | 488.4 | 122.9 | 223.1 |
|  | 5 | 249.6 | 3833.7 | 809.8 | 701.2 | 245.5 | 250.5 |
|  | 5.5 | 322.6 | 12101.7 | 2543 | 859.3 | 498.6 | 280.6 |

Table S14 - Griseofulvin, itraconazole, posaconazole, and isavuconazole VD_ss_ in liters using the Oie-Tozer, Rodgers-Rowland (tissue-specific Kp and muscle Kp), GastroPlus, Korzekwa-Nagar, and TCM-New methods at specific logP values.

|  | **LogP** | **Oie-Tozer** | **Rodgers-Rowland**  **(tissue specific Kp)** | **Rodgers-Rowland (muscle Kp only)** | **GastroPlus** | **Korzekwa-Nagar** | **TCM-New** |
| --- | --- | --- | --- | --- | --- | --- | --- |
| **Griseofulvin** | 2.11 | 92.3 | 213.4 | 56.3 | 124.8 | 90.5 | 115.5 |
|  | 3.53 | 152.0 | 2089.9 | 449.7 | 437.9 | 352.7 | 182.8 |
|  | 3.566 | 154.7 | 2269.2 | 487.3 | 448.7 | 370.9 | 184.8 |
| **Itraconazole** | 4.893 | 193.2 | 606.5 | 133.0 | 374.0 | 30.2 | 220.8 |
|  | 5.66 | 285.8 | 3500.3 | 739.6 | 753.8 | 76.3 | 262.8 |
|  | 6.888 | 539.5 | 59017.6 | 12377.4 | 1146.3 | 415.8 | 347.0 |
| **Posaconazole** | 4.405 | 201.8 | 1950.1 | 415.3 | 557.9 | 131.0 | 198.0 |
|  | 5.36 | 328.8 | 17498.5 | 3674.6 | 862.0 | 342.2 | 246.7 |
|  | 6.716 | 664.2 | 396968.1 | 83221.0 | 1194.6 | 2396.1 | 335.2 |
| **Isavuconazole** | 3.56 | 121.0 | 148.8 | 37.3 | 119.7 | 39.9 | 178.4 |
|  | 3.619 | 124.6 | 169.0 | 41.6 | 133.8 | 37.3 | 175.3 |
|  | 4.934 | 241.4 | 3294.6 | 696.8 | 676.4 | 223.8 | 246.7 |

Table S15 – Values for VD_ss_ prediction fold errors, absolute average (AAFE), and average fold error (AFE) per method for griseofulvin, itraconazole, posaconazole, and isavuconazole across different sources of logP values (ADMET Predictor, literature, HPLC-based). AAFE and AFE values were calculated using Equations 15 and 16. Table here supplements Table 5.

|  | **ADMET Predictor** | **Oie-Tozer** | | | **Rodgers-Roland**  **(tissue-specific Kp)** | | | | **Rodgers-Roland**  **(muscle only Kp)** | | | | **GastroPlus** | | | | **Korzekwa-Nagar** | | | **TCM-New** | | | |
| --- | --- | --- | --- | --- | --- | --- | --- | --- | --- | --- | --- | --- | --- | --- | --- | --- | --- | --- | --- | --- | --- | --- | --- |
| **Drug** | **LogP** | **Fold error** | **AFE** | **AAFE** | **Fold error** | **AFE** | **AAFE** | **Fold error** | | **AFE** | **AAFE** | **Fold error** | | **AFE** | **AAFE** | **Fold error** | | **AFE** | **AAFE** | | **Fold error** | **AFE** | **AAFE** |
| Griseofulvin | 2.511 | 0.86 | 0.49 | 2.03 | 1.99 | 1.54 | 1.54 | 0.53 | | 0.36 | 2.77 | 1.17 | | 0.82 | 1.22 | 0.85 | | 0.21 | 4.81 | | 1.08 | 0.59 | 1.71 |
| Itraconazole | 4.893 | 0.24 |  |  | 0.76 |  |  | 0.17 | |  |  | 0.47 | |  |  | 0.04 | |  |  |  | 0.28 |  |  |
| Posaconazole | 4.405 | 0.69 |  |  | 6.63 |  |  | 1.41 | |  |  | 1.90 | |  |  | 0.45 | |  |  |  | 0.67 |  |  |
| Isavuconazole | 3.619 | 0.41 |  |  | 0.56 |  |  | 0.14 | |  |  | 0.44 | |  |  | 0.13 | |  |  |  | 0.59 |  |  |
|  | **Literature** | **Oie-Tozer** | | | **Rodgers-Roland**  **(tissue-specific Kp)** | | | | **Rodgers-Roland**  **(muscle only Kp)** | | | | **GastroPlus** | | | | **Korzekwa-Nagar** | | | **TCM-New** | | | |
| **Drug** | **LogP** | **Fold error** | **AFE** | **AAFE** | **Fold error** | **AFE** | **AAFE** | **Fold error** | | **AFE** | **AAFE** | **Fold error** | | **AFE** | **AAFE** | **Fold error** | | **AFE** | **AAFE** | | **Fold error** | **AFE** | **AAFE** |
| Griseofulvin | 3.53 | 1.42 | 0.69 | 1.45 | 19.53 | 7.06 | 7.06 | 4.20 | | 1.56 | 1.56 | 4.09 | | 1.45 | 1.45 | 3.30 | | 0.46 | 2.17 | | 1.71 | 0.72 | 1.39 |
| Itraconazole | 5.66 | 0.36 |  |  | 4.38 |  |  | 0.92 | |  |  | 0.94 | |  |  | 0.10 | |  |  |  | 0.33 |  |  |
| Posaconazole | 5.36 | 1.12 |  |  | 59.52 |  |  | 12.50 | |  |  | 2.93 | |  |  | 1.16 | |  |  |  | 0.84 |  |  |
| Isavuconazole | 3.56 | 0.40 |  |  | 0.49 |  |  | 0.12 | |  |  | 0.39 | |  |  | 0.12 | |  |  |  | 0.58 |  |  |
|  | **HPLC-based** | **Oie-Tozer** | | | **Rodgers-Roland**  **(tissue-specific Kp)** | | | | **Rodgers-Roland**  **(muscle only Kp)** | | | | **GastroPlus** | | | | **Korzekwa-Nagar** | | | **TCM-New** | | | |
| **Drug** | **LogP** | **Fold error** | **AFE** | **AAFE** | **Fold error** | **AFE** | **AAFE** | **Fold error** | | **AFE** | **AAFE** | **Fold error** | | **AFE** | **AAFE** | **Fold error** | | **AFE** | **AAFE** | | **Fold error** | **AFE** | **AAFE** |
| Griseofulvin | 3.566 | 1.45 | 1.15 | 1.15 | 21.21 | 69.17 | 69.17 | 4.55 | | 14.62 | 14.62 | 4.19 | | 2.71 | 2.71 | 3.47 | | 1.81 | 1.81 | | 1.73 | 0.91 | 1.10 |
| Itraconazole | 6.888 | 0.67 |  |  | 73.77 |  |  | 15.47 | |  |  | 1.43 | |  |  | 0.52 | |  |  |  | 0.43 |  |  |
| Posaconazole | 6.716 | 2.26 |  |  | 1350.23 |  |  | 283.06 | |  |  | 4.06 | |  |  | 8.15 | |  |  |  | 1.14 |  |  |
| Isavuconazole | 4.934 | 0.79 |  |  | 10.84 |  |  | 2.29 | |  |  | 2.22 | |  |  | 0.74 | |  |  |  | 0.81 |  |  |

Table S16 - Values for VD_ss_ prediction fold errors, absolute average (AAFE), and average fold error (AFE) per method across the four model drugs (griseofulvin, itraconazole, posaconazole, and isavuconazole) and their logP values. AAFE and AFE values were calculated using Equations 15 and 16.Table here supplements Table 6.

|  |  | **Oie-Tozer** | | | **Rodgers-Roland**  **(tissue-specific Kp)** | | | **Rodgers-Roland**  **(muscle only Kp)** | | | **GastroPlus** | | | **Korzekwa-Nagar** | | | **TCM-New** | | |
| --- | --- | --- | --- | --- | --- | --- | --- | --- | --- | --- | --- | --- | --- | --- | --- | --- | --- | --- | --- |
| **Drug** | **LogP** | **Fold error** | **AFE** | **AAFE** | **Fold error** | **AFE** | **Fold error** | **Fold error** | **Fold error** | **AAFE** | **Fold error** | **AFE** | **AAFE** | **Fold error** | **AFE** | **AAFE** | **Fold error** | **AFE** | **AAFE** |
| Griseofulvin | 2.511 | 0.86 | 1.21 | 1.21 | 1.99 | 9.38 | 9.38 | 0.53 | 6.35 | 6.35 | 1.17 | 2.71 | 2.71 | 0.85 | 2.13 | 2.13 | 1.08 | 1.47 | 1.47 |
|  | 3.53 | 1.42 |  |  | 19.53 |  |  | 4.20 |  |  | 4.09 |  |  | 3.30 |  |  | 1.71 |  |  |
|  | 3.566 | 1.45 |  |  | 21.21 |  |  | 115.68 |  |  | 4.19 |  |  | 3.47 |  |  | 1.73 |  |  |
|  |  | **Oie-Tozer** | | | **Rodgers-Roland**  **(tissue-specific Kp)** | | | **Rodgers-Roland**  **(muscle only Kp)** | | | **GastroPlus** | | | **Korzekwa-Nagar** | | | **TCM-New** | | |
| **Drug** | **LogP** | **Fold error** | **AFE** | **AAFE** | **Fold error** | **AFE** | **AAFE** | **Fold error** | **AFE** | **AAFE** | **Fold error** | **AFE** | **AAFE** | **Fold error** | **AFE** | **AAFE** | **Fold error** | **AFE** | **AAFE** |
| Itraconazole | 4.893 | 0.24 | 0.39 | 2.58 | 0.76 | 6.25 | 6.25 | 0.17 | 1.33 | 1.33 | 0.47 | 0.86 | 1.17 | 0.04 | 0.12 | 8.11 | 0.28 | 0.34 | 2.94 |
|  | 5.66 | 0.36 |  |  | 4.38 |  |  | 0.92 |  |  | 0.94 |  |  | 0.10 |  |  | 0.33 |  |  |
|  | 6.888 | 0.67 |  |  | 73.77 |  |  | 15.47 |  |  | 1.43 |  |  | 0.52 |  |  | 0.43 |  |  |
|  |  | **Oie-Tozer** | | | **Rodgers-Roland**  **(tissue-specific Kp)** | | | **Rodgers-Roland**  **(muscle only Kp)** | | | **GastroPlus** | | | **Korzekwa-Nagar** | | | **TCM-New** | | |
| **Drug** | **LogP** | **Fold error** | **AFE** | **AAFE** | **Fold error** | **AFE** | **AAFE** | **Fold error** | **AFE** | **AAFE** | **Fold error** | **AFE** | **AAFE** | **Fold error** | **AFE** | **AAFE** | **Fold error** | **AFE** | **AAFE** |
| Posaconazole | 4.41 | 0.69 | 1.20 | 1.20 | 6.63 | 81.08 | 81.08 | 1.41 | 17.10 | 17.10 | 1.90 | 2.83 | 2.83 | 0.45 | 1.62 | 1.62 | 0.67 | 0.86 | 1.16 |
|  | 5.36 | 1.12 |  |  | 59.52 |  |  | 12.50 |  |  | 2.93 |  |  | 1.16 |  |  | 0.84 |  |  |
|  | 6.72 | 2.26 |  |  | 1350.23 |  |  | 283.06 |  |  | 4.06 |  |  | 8.15 |  |  | 1.14 |  |  |
|  |  | **Oie-Tozer** | | | **Rodgers-Roland**  **(tissue-specific Kp)** | | | **Rodgers-Roland**  **(muscle only Kp)** | | | **GastroPlus** | | | **Korzekwa-Nagar** | | | **TCM-New** | | |
| **Drug** | **LogP** | **Fold error** | **AFE** | **AAFE** | **Fold error** | **AFE** | **AAFE** | **Fold error** | **AFE** | **AAFE** | **Fold error** | **AFE** | **AAFE** | **Fold error** | **AFE** | **AAFE** | **Fold error** | **AFE** | **AAFE** |
| Isavuconazole | 3.619 | 0.41 | 0.51 | 1.98 | 0.56 | 1.43 | 1.43 | 0.14 | 0.34 | 2.96 | 0.44 | 0.73 | 1.37 | 0.13 | 0.23 | 4.38 | 0.59 | 0.65 | 1.54 |
|  | 3.56 | 0.40 |  |  | 0.49 |  |  | 0.12 |  |  | 0.39 |  |  | 0.12 |  |  | 0.58 |  |  |
|  | 4.934 | 0.79 |  |  | 10.84 |  |  | 2.29 |  |  | 2.22 |  |  | 0.74 |  |  | 0.81 |  |  |
